# Sirtuin 1 deficiency mediates chronic kidney disease-induced inflammaging cardiovascular calcification

**DOI:** 10.1186/s43556-026-00488-3

**Published:** 2026-07-07

**Authors:** Li Xu, Yidan Zheng, Ming Liu, Bowen Deng, Xingyu Qian, Chen Jiang, Yuqi Liu, Pengning Fan, Zhenqi Rao, Ming Chen, Zhe Chen, Zhejun Cai, Nianguo Dong, Da Zhu, Fei Li

**Affiliations:** 1 Department of Cardiovascular Surgery, Union Hospital, Tongji Medical College, Huazhong University of Science and Technology, 1277 Jiefang Ave., Wuhan, 430022 China; 2 Department of Nephrology, Union Hospital, Tongji Medical College, Huazhong University of Science and Technology, 1277 Jiefang Ave., Wuhan, 430022 China; 3 Department of Cardiac Surgery, Yunnan Fuwai Cardiovascular Hospital, Kunming Medical University, 528 Shahebei Road, Kunming, 65000 China; 4 Department of Cardiology, The Second Affiliated Hospital, Zhejiang University School of Medicine, 88 Jiefang Road, Hangzhou, Zhejiang 310009 China

**Keywords:** Chronic kidney disease, Aortic valve stenosis, Sirtuin 1, NLR Family Pyrin domain-containing 3 protein, Semaglutide

## Abstract

Chronic kidney disease (CKD) markedly accelerates calcific aortic valve disease (CAVD), yet the underlying mechanisms and therapeutic targets remain poorly defined. Here, we integrate population-scale analyses, single-cell transcriptomics, genetic inference and functional experiments to identify a central role for Sirtuin 1 (SIRT1) in CKD-associated aortic valve calcification. Analysis of UK Biobank data linked CKD to accelerated ageing and increased aortic stenosis risk. Single-cell RNA sequencing of human aortic valves identified SIRT1 downregulation and NLRP3 pathway activation specifically in myofibroblast valve interstitial cells (VICs), coupled with enhanced senescence and osteogenic programmes. Consistently, genetic analyses, including eQTL-based Mendelian randomization, supported an inverse association between SIRT1 expression and CAVD risk. SIRT1 deficiency was associated with enhanced glycolysis, increased NF-κB activation and NLRP3 inflammasome signalling, accompanied by augmented osteogenic differentiation and calcification of VICs. Pharmacological or genetic inhibition of NLRP3 attenuated valve calcification in vivo, establishing the SIRT1-NF-κB-NLRP3 axis as a critical pathway linking CKD to CAVD. Finally, screening of anti-diabetic compounds identified semaglutide as a potent modulator that restores the SIRT1/NLRP3 balance and alleviates calcification in vitro and in vivo. These findings define a metabolic-inflammatory coupling mechanism underlying CKD-induced CAVD and highlight SIRT1 as a therapeutic target. Modulation of the SIRT1-NLRP3 axis, particularly by semaglutide, may represent a promising strategy for preventing valve calcification.

**Supplementary Information:**

The online version contains supplementary material available at 10.1186/s43556-026-00488-3.

## Introduction

Patients with chronic kidney disease (CKD) carry high cardiovascular risk, including vascular calcification, heart failure, coronary artery disease, arrhythmias, and sudden death [1]. While vascular calcification in CKD has been investigated, valvular calcification has received less mechanistic attention, despite its strong association with CKD and its substantial clinical consequences [2, 3]. Aortic valve calcification, a key pathological substrate of calcific aortic valve disease (CAVD), is disproportionately observed in CKD populations and is associated with accelerated disease progression in patients with advanced kidney dysfunction, particularly end-stage renal disease (ESRD) [4, 5]. These observations suggest that CKD does not merely coexist with CAVD but may actively accelerate valvular calcific remodeling.

The prevalence and severity of valvular calcification increase progressively as kidney function declines. Mitral and aortic valve calcification are more frequently observed in advanced CKD and are closely linked to disturbances in calcium-phosphate metabolism, dysregulation of matrix Gla protein (MGP), imbalance of the OPG/RANK/RANKL axis, and alterations in fetuin A and FGF23/Klotho signaling [6, 7]. The burden of valvular calcification increases with declining kidney function, with more advanced CKD and lower eGFR consistently associated with a higher prevalence and greater severity of valvular calcification [4, 5]. These epidemiological and clinical findings highlight a disease continuum in which impaired renal function, systemic metabolic imbalance, and local valvular remodeling converge to promote calcific valve disease.

CAVD is now recognized as an active, cell-mediated process rather than passive calcium deposition. Its pathogenesis involves chronic inflammation, disordered mineral metabolism, osteogenic transdifferentiation of valvular interstitial cells (VICs), and reduced activity of endogenous calcification inhibitors, all of which may be intensified in the CKD milieu [8–10]. However, the molecular mechanisms linking CKD-associated inflammatory and metabolic stress to VIC osteogenic reprogramming remain incompletely defined.

Sirtuin 1 (SIRT1), a nuclear NAD + -dependent deacetylase and the human orthologue of yeast Sir2, has been implicated in longevity, metabolic homeostasis, and protection against cellular senescence [11, 12]. Mechanistically, SIRT1 deacetylates RELA/NF-κB p65 and thereby suppresses pro-inflammatory cytokine expression [13]. It also regulates inflammaging by mitigating oxidative stress, mitochondrial injury, and chronic inflammatory activation [14]. In vascular calcification, SIRT1 downregulation has been associated with calcific remodeling, whereas SIRT1 activation preserves mitochondrial homeostasis and attenuates calcification [15–17]. Nevertheless, whether SIRT1 mediates CKD-induced aortic valve calcification, and through which downstream inflammatory and metabolic mechanisms it may act in VICs, remains largely unexplored.

Here, we hypothesized that SIRT1 serves as a critical molecular link between CKD-associated metabolic-inflammatory stress and aortic valve calcification. To test this hypothesis, we integrated population-level evidence from UK Biobank, human valve single-cell RNA sequencing, genetic analyses, and complementary in vitro and in vivo validation models. Our analyses identified reduced SIRT1 expression and activation of the NLRP3 inflammasome in myofibroblast-like VICs, accompanied by enhanced cellular senescence and osteogenic reprogramming. Mechanistically, SIRT1 deficiency promoted glycolysis-dependent activation of the NF-κB-NLRP3 inflammatory axis, thereby driving VIC calcification. Pharmacological and genetic restoration of this pathway, including semaglutide-mediated intervention, attenuated calcific remodeling. These findings define a SIRT1-centered inflammatory-metabolic mechanism underlying CKD-associated aortic valve calcification and suggest a potential therapeutic strategy for this high-risk patient population.

## Results

### CKD promoted aging phenotype and aortic valve calcification

**Fig. 1:**
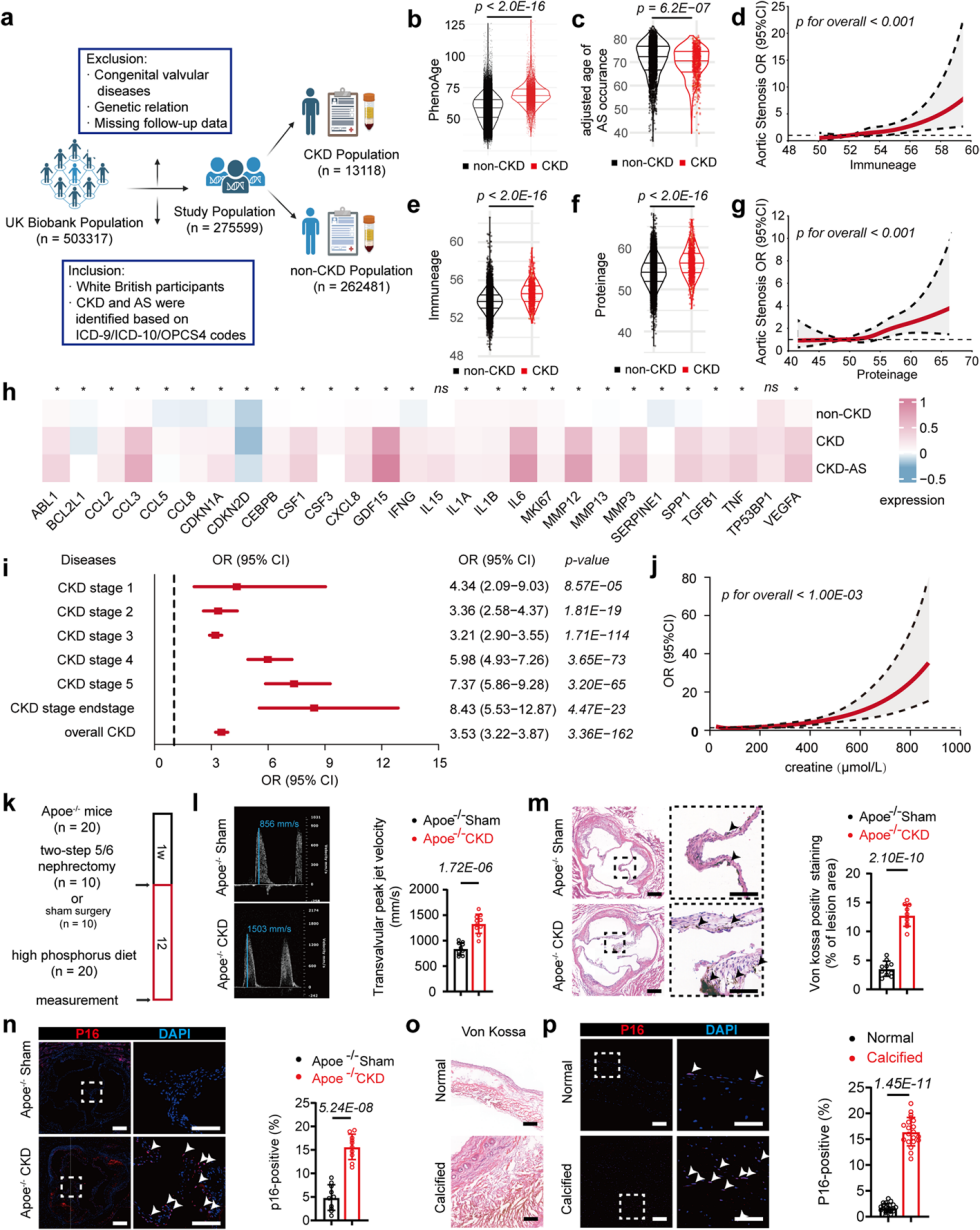
Chronic kidney disease promoted senescence phenotype and calcification in aortic valves. **a** Strategy for establishing the UKB population cohort, including exclusion or access criteria and population composition. **b** Chronic kidney disease (CKD) population (*n* = 13,118) exhibited significantly elder PhenoAge than non-CKD population (*n* = 262,481) in UKB. **c** CKD population (*n* = 13,118) exhibited significantly younger adjusted age of AS occurrence than non-CKD population in UKB. **d** Restricted cubic spline regression of immuneage and AS OR. **e** CKD population exhibited significantly elder immuneage than non-CKD population in UKB. **f** CKD population exhibited significantly elder proteinage than non-CKD population in UKB. **g** Restricted cubic spline regression of proteinage and AS OR. **h** Proteomic analyses showed significant upregulation of aging-related indicators in the CKD population, more prominent in the group suffering from AS. **i** Odds ratios (ORs) of CKD by stage on the onset of AS. **j** Restricted cubic spline regression of plasma creatinine level and AS OR. **k** Schematic diagram of establishing and monitoring chronic kidney disease (CKD) model. **l** Echocardiography showed that *Apoe*^*−/−*^ CKD mice had significantly higher transvalvular peak jet velocity than *Apoe*^*−/−*^ sham mice. *n* = 10 per group. **m** Representative images of von Kossa staining of calcium in aortic valves of mice. *n* = 10 per group. Calcium deposition was significantly increased in aortic valves of *Apoe*^*−/−*^ CKD mice compared with that in *Apoe*^*−/−*^ sham mice. *n* = 10 per group. Scale bar = 250 μm. **n** Representative images of immunofluorescence staining for p16 (red) in aortic valves of *Apoe*^*−/−*^ mice. DAPI (blue) was used for nuclear counterstaining. *n* = 10 per group. *Apoe*^*−/−*^ CKD mice had significantly higher p16 expression than *Apoe*^*−/−*^ sham mice. *n* = 10 per group. Scale bar = 250 μm. **o** and **p** Representative images of von Kossa staining and immunofluorescence staining for P16 (red) in normal and calcified human aortic valves. DAPI (blue) was used for nuclear counterstaining. *n* = 10 per group. Calcified human aortic valves had higher level of P16 than that in normal control, *n* = 20 per group. Scale bar = 100 μm. Created with BioRender.com

To determine whether CKD is associated with accelerated biological aging and increased risk of aortic valve calcification, we first performed epidemiological analyses in the UK Biobank cohort and validated the findings in a CKD mouse model. A total of 275,599 unrelated White British participants from the UK Biobank were included in our study to evaluate the progression from CKD to aging phenotypes and aortic stenosis (AS), after excluding individuals with congenital valvular diseases (Fig. 1a). Disease definitions were based on ICD-9, ICD-10 and OPCS4 codes, as detailed in Table S1. CKD patients posed elder phenoages and younger adjusted AS occurrence ages (Fig. 1b-g). Plasma proteomic analysis revealed that CKD patients present higher aging-related biomarkers in their plasma, which is more prominent in AS patients (Fig. 1h). A significant increase in the odds ratio of AS onset was observed as CKD progressed in specific subgroup analyses (Fig. 1i). Restricted cubic spline indicated that the risk of AS was significantly increased with elevated creatinine levels (Fig. 1j).

To further elucidate whether CKD promotes aortic valve calcification through the aging-related pathway, eight-week-old male *Apoe*^*−/−*^ mice were randomly divided into 5/6 nephrectomy group (*n* = 10) or sham surgery group (*n* = 10) and then fed with high-phosphate fodder for 12 weeks to establish an aortic valve calcification model (Fig. 1k). Echocardiography revealed an obvious increase in transvalvular peak jet velocity in CKD mice compared with the sham group (Fig. 1l). Moreover, von Kossa staining showed more calcium deposition in aortic valve leaflets of CKD mice (Fig. 1m). Meanwhile, immunofluorescence staining revealed a drastic increase in p16 expression in CKD mice (Fig. 1n). Immunofluorescence staining and von Kossa staining showed that the expression of aging-related P16 significantly increased in calcified aortic valve tissues of CKD patients compared with those of controls (Fig. 1o, p). These results indicated that CKD might promote senescence phenotype and calcification in aortic valve.

### SIRT1 expression was decreased in CKD patients and* Apoe*^*−/−*^mice

**Fig. 2:**
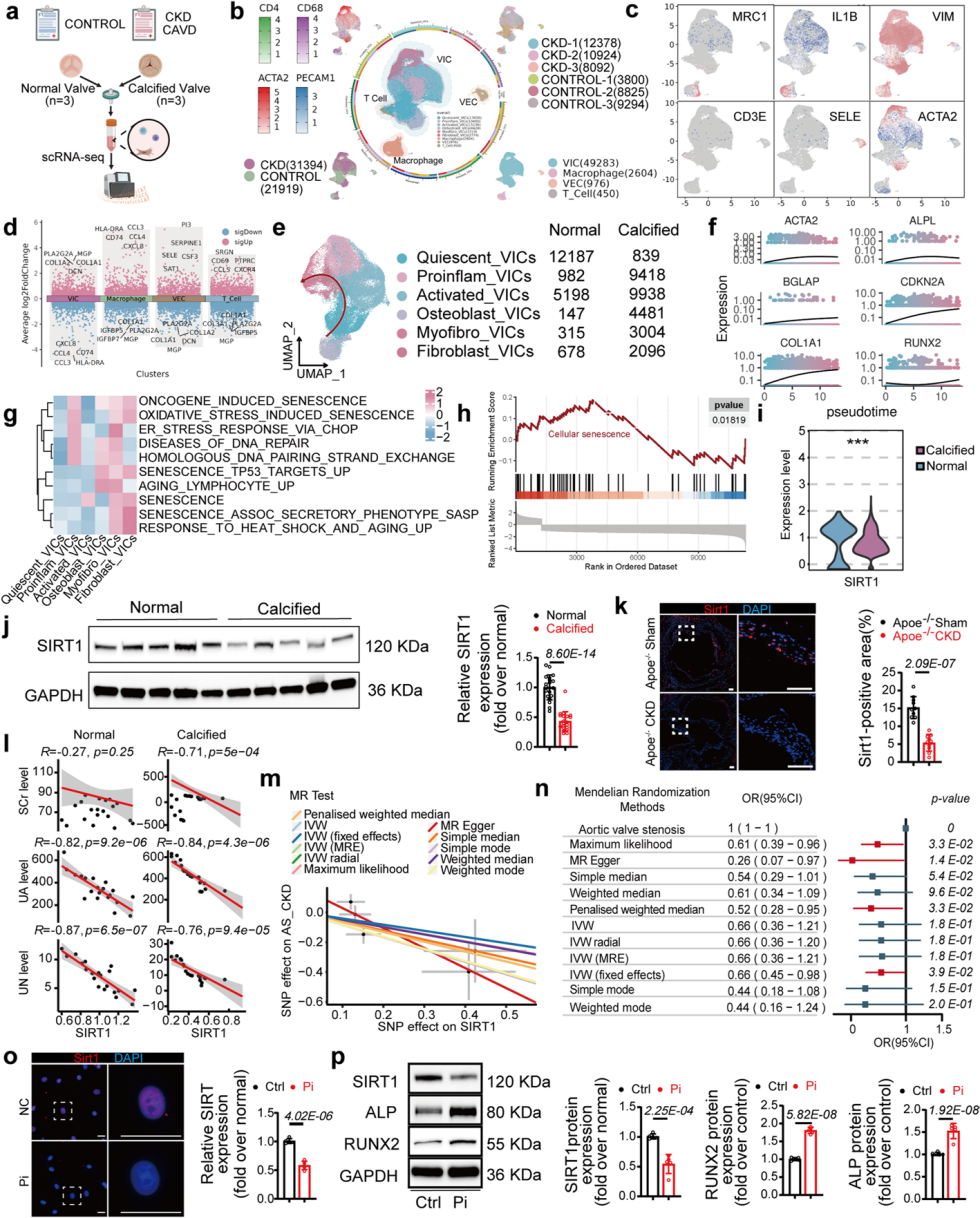
Single-cell RNA sequence revealed chronic kidney disease decreased the expression of SIRT1 in aortic valves. **a** Schematic diagram of single cell RNA (scRNA) sequencing in normal valves from control and calcified valves from CKD patients. *n* = 3 per group. **b** ScRNA atlas of aortic valves from control and CKD patients. **c** Major markers for cell types in aortic valves. **d** Differential gene expression volcano plots of the four major aortic valve cell subclusters. **e** Trajectory analysis of various subpopulations of VICs from normal versus calcified aortic valves, with myofibroblasts-VICs emerging as a critical and late state in the calcification process. The number of cells in each VIC subpopulation is listed aside. **f** Changes in aging and calcification-related genes with trajectory progression. **g** GSVA pathway enrichment analysis revealed that senescence-related pathways are upregulated in myofibroblast-VICs. **h** GSEA pathway enrichment analysis revealed that the cellular senescence pathway significantly upregulated in myofibroblast-VICs. **i** Violin plot of SIRT1 levels in myofibroblast-VICs. **j** Representative western blot band of SIRT1 expression in calcified and normal human aortic valves. Calcified human aortic valves had significantly lower level of SIRT1 expression than control. *n* = 20 per group. **k** Representative images of immunofluorescence staining for Sirt1 (red) in aortic valves of *Apoe*^*−/−*^ mice. DAPI (blue) was used for nuclear counterstaining. *n* = 10 per group. Sirt1 expression was significantly decreased in *Apoe*^*−/−*^ CKD mice compared to *Apoe*^*−/−*^ sham mice. *n* = 10 per group. Scale bar = 100 μm. **l** Regression between serum creatinine level, uric acid level, urea nitrogen level and expression of SIRT1 in aortic valves. *n* = 20 per group. **m** Scatterplot of expression quantitative trait loci (eQTL) Mendelian randomization (MR) analysis on SIRT1 expression levels and the occurrence of AS in CKD patients. **n** Forest plot of the detailed correlation of SIRT1 expression levels on AS occurrence from the results obtained from eQTL-MR. **o** Representative images of immunofluorescence staining and quantification analysis showed that high phosphorous treatment VICs had significantly lower expression of SIRT1 (red), DAPI (blue) was used for nuclear counterstaining. *n* = 5 per group. Scale bar = 25 μm. **p** Representative western blot band of SIRT1, ALP, RUNX2 expression in human valve interstitial cells (VICs) treated with hyperphosphate culture medium for 72 h. *n* = 5 per group. Densitometric quantification showed that hyperphosphate decreased SIRT1 expression, increased ALP and RUNX2 expression in VICs. *n* = 5 per group. Created with BioRender.com

To reveal the specific mechanism mediated the CKD and aortic valve calcification, a set of scRNA was conducted on 3 calcified aortic valves from CKD patients and 3 normal aortic valves from control patients (Fig. 2a and Fig. S1a-1f). Four major primary cell types were identified by previously reported markers and differentially expressed genes (DEGs). VICs were further divided into 6 subtypes defined by cell functions (Fig. 2b-d). Cell cluster annotation and function enrichment were shown in Figs. S2-3 and Fig. S4a-4f in detail. Higher proportions of proinflammatory-VICs, osteoblast-VICs, fibroblast-VICs and myofibroblast-VICs were observed in CKD-induced calcified aortic valves (Fig. 2e). Trajectory analysis suggested that quiescent-VICs gradually shifted to myofibroblast VICs during the CAVD process, accompanied by upregulation of osteogenic genes (*ACTA2, BGLAP, COL1A1, RUNX2*) and senescent gene expression (*ALPL, CDKN2A*) (Fig. 2f). GSVA results showed that senescence-related pathways were significantly up-regulated in CKD-induced calcified valve VICs, with myofibroblast-VICs showing the most pronounced trend of elevation (Fig. 2g), which was not observed in other VICs subtypes. Specific GSEA analysis confirmed the up-regulation of cellular senescence pathway in myofibroblast-VICs from CKD patients (Fig. 2h and Fig. S5a-5e). SIRT1 expression level significantly declined in myofibroblast-VICs in calcified valves compared to normal valves (Fig. 2i), and this feature was even more pronounced in CKD-associated CAVD valve tissues (Fig. S6a-c). In the valve endothelial cell (VEC) subset, NOS3 and SIRT1 exhibited a strong positive co-expression, with this correlation being more pronounced in CKD patients (Fig. S6d-f).

Western blot analysis of valve tissue showed that the expression of SIRT1 significantly decreased in calcified aortic valve tissues of CKD patients compared with those of controls (Fig. 2j). Immunofluorescence staining showed that the expression of Sirt1 significantly decreased in aortic valves of CKD mice compared with those of sham controls (Fig. 2k). Regression analysis with clinical tests showed that SIRT1 expression levels significantly showed a negative correlation with creatinine, uric acid, and urea nitrogen levels (Fig. 2l). In vitro investigation received conserving results. Immunofluorescence staining also obtained a result of SIRT1 expression level decrease in VICs treated to hyperphosphate medium (Fig. 2o). After we exposed human aortic VICs to hyperphosphate medium, western blot and immunofluorescence staining analysis both showed that the expression of SIRT1 significantly declined, along with ALP and runt-related transcription factor 2 (RUNX2) up-regulation (Fig. 2p). Taken together, these results identify SIRT1 downregulation, particularly in myofibroblast-VICs, as a key molecular feature associated with CKD-induced valve calcification and cellular senescence.

### Genetic association of SIRT1 with CAVD

In order to determine whether the genetic variations at *SIRT1* were associated with aortic stenosis and worse outcome, a large-scale cohort GWAS was conducted (*n* = 13,118, with 832 aortic stenosis cases, genotyping methods were described in Fig. S7a). A total of 430 variants in *SIRT1* gene were nominally significant (*P* < 0.05) for aortic stenosis (Fig. S7b-7c). The top 10 variants are shown in Fig. S7d, with associations reaching a minimum *P* value of 6.9E-04 or an odds ratio of up to 1.95 for aortic stenosis. Expression Quantitative Trait Loci Mendelian randomization analysis (eQTL-MR) demonstrated that *SIRT1* expression in valve-related tissue negatively correlated to odds ratio of aortic stenosis occurrence, significant in maximum likelihood, MR Egger, penalized weighted median and IVW fixed effects method (Fig. 2m-n and Table S4). The findings indicated high *SIRT1* expression related to the slower progression to severe aortic stenosis.

### SIRT1 intervention influenced aortic valve calcification in vivo and in vitro

To functionally validate the role of SIRT1 in CKD-induced aortic valve calcification, we modulated SIRT1 expression in vivo and in vitro and assessed its effects on calcification and senescence phenotypes. We generated CKD condition in *Apoe*^*−/−*^ mice (*n* = 10), *Sirt1*^*+/−*^*Apoe*^*−/−*^ mice (*n* = 10), *Apoe*^*−/−*^ mice supplied with resveratrol (a SIRT1 specific agonist) (*n* = 10), myofibroblast SIRT1 knockout *Apoe*^*−/−*^ mice (*Sirt1*^*mfKO*^ *Apoe*^*−/−*^ mice, *n* = 10), and myofibroblast SIRT1 overexpressing *Apoe*^*−/−*^ mice (*Sirt1*^*mfOV*^ *Apoe*^*−/−*^ mice, *n* = 10), and fed them with high-phosphate fodder for 12 weeks to establish aortic valve calcification. Detailed construction strategies for gene-edited animals with validation of gene identification were revealed in the Figs. S8-9. Biochemical examinations showed no significant difference between gene intervention models (Fig. S10a-10 l).

**Fig. 3:**
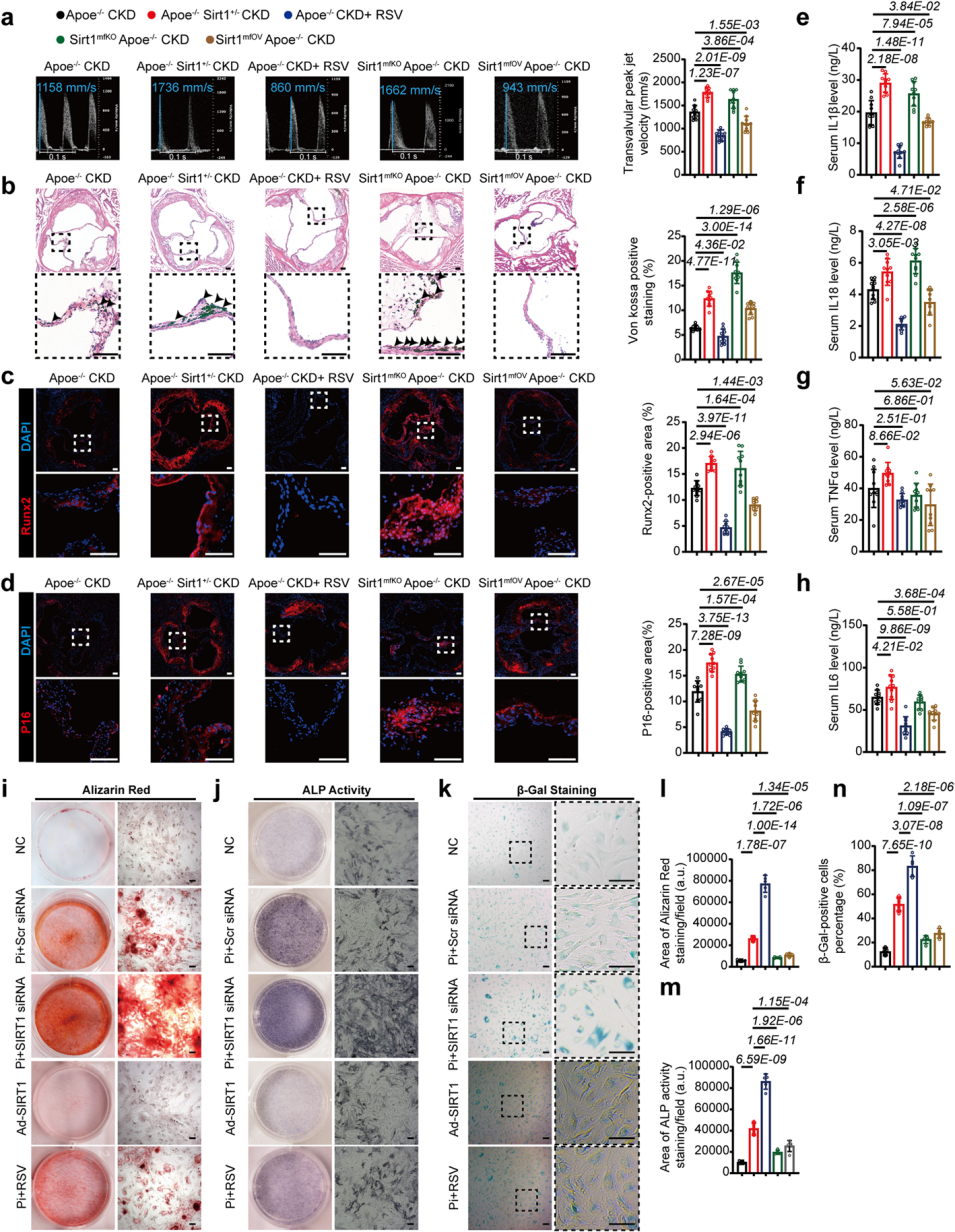
Intervention of the SIRT1 influenced aortic valve calcification and senescence in vivo *and* in vitro. **a** Echocardiography showed that *Sirt1*^*+/−*^ *Apoe*^*−/−*^ and *Sirt1*^*mfKO*^ *Apoe*^*−/−*^ mice had significantly higher transvalvular peak jet velocity, and *Sirt1*^*mfOV*^ *Apoe*^*−/−*^ mice and those supplied with resveratrol had significantly lower transvalvular peak jet velocity than *Apoe*^*−/−*^ CKD mice. *n* = 10 per group. **b** Representative images of von Kossa staining of calcium in aortic valves of CKD mice. *n* = 10 per group. Calcium deposition was significantly increased in aortic valves of *Sirt1*^*+/−*^ *Apoe*^*−/−*^ and *Sirt1*^*mfKO*^ *Apoe*^*−/−*^ mice and decreased in *Sirt1*^*mfOV*^ *Apoe*^*−/−*^ mice and those supplied with resveratrol, compared with *Apoe*^*−/−*^ CKD mice. *n* = 10 per group. Scale bar = 100 μm. **c** Representative images of immunofluorescence staining and quantification analysis showed that *Sirt1*^*+/−*^ *Apoe*^*−/−*^ and *Sirt1*^*mfKO*^ *Apoe*^*−/−*^ mice had significantly higher expression of Runx2 (red), and *Sirt1*^*mfOV*^ *Apoe*^*−/−*^ mice and those supplied with resveratrol had significantly lower expression of Runx2 (red) than *Apoe*^*−/−*^ CKD mice, DAPI (blue) was used for nuclear counterstaining. *n* = 10 per group. Scale bar = 100 μm. **d** Representative images of immunofluorescence staining and quantification analysis showed that Sirt1^*+/−*^ *Apoe*^*−/−*^ and *Sirt1*^*mfKO*^ *Apoe*^*−/−*^ mice had significantly higher expression of P16 (red), and *Sirt1*^*mfOV*^ *Apoe*^*−/−*^ mice and those supplied with resveratrol had significantly lower expression of P16 (red) than *Apoe*^*−/−*^ CKD mice, DAPI (blue) was used for nuclear counterstaining. *n* = 10 per group. Scale bar = 100 μm. **e**, **f**, **g**, and **h** Quantification showed that *Sirt1*^*+/−*^ *Apoe*^*−/−*^ and *Sirt1*^*mfKO*^ *Apoe* ^*−/−*^ mice had higher level of inflammatory cytokines, and *Sirt1*^*mfOV*^ *Apoe*^*−/−*^ mice and those supplied with resveratrol had lower level of inflammatory cytokines. *n* = 10 per group. **i** and **l** Representative images of staining and quantification analysis showed down-regulating SIRT1 led to elevating of alizarin red level. *n* = 5 per group. Scale bar = 25 μm. **j** and **m** Representative images of staining and quantification analysis showed down-regulating SIRT1 led to elevating of ALP activity level. *n* = 5 per group. Scale bar = 25 μm. **k** and **n** Representative images of staining and quantification analysis showed down-regulating SIRT1 led to elevating of β-Gal staining level. *n* = 5 per group. Scale bar = 25 μm

After 12 weeks of high-phosphate feeding, transvalvular peak jet velocity of aortic valve was significantly higher in *Sirt1*^*+/−*^*Apoe*^*−/−*^ and *Sirt1*^*mfKO*^* Apoe*^*−/−*^ mice, lower in *Sirt1*^*mfOV*^* Apoe*^*−/−*^ mice and those supplied with resveratrol, compared with *Apoe*^*−/−*^ group (Fig. 3a). Von Kossa staining showed that *Sirt1*^+/−^*Apoe*^*−/−*^ mice had a larger area of calcification in aortic valve than *Apoe*^*−/−*^ mice, while resveratrol had a smaller calcification (Fig. 3b). Calcium-specific Alizarin Red S staining yielded results consistent with von Kossa staining (Fig. S9d). In addition, Runx2 and p16 were simultaneously elevated in *Sirt1*^+/−^ *Apoe*^*−/−*^ mice and *Sirt1*^*mfKO*^* Apoe*^*−/−*^ mice and down-regulated in *Sirt1*^*mfOV*^ *Apoe*^*−/−*^ mice and those supplied with resveratrol, than *Apoe*^*−/−*^ mice (Fig. 3c, d). Inflammatory cytokine levels in mice plasma were also detected by ELISA. The results showed that mice with systemic or myofibroblast-specific SIRT1 gene deficiency presented a significant up-regulation of inflammatory cytokine levels compared to *Apoe*^*−/−*^ mice, while those with systemic or myofibroblast-specific SIRT1 activation presented the inverse results (Fig. 3e, f, g, h). To further clarify the specific role of SIRT1 in valve calcification under CKD conditions, we supplemented control data from SIRT1 knockout and overexpression models under non-CKD (sham) conditions (Fig. S11). The results showed that under non-CKD conditions, SIRT1 alterations had only modest effects on valve calcification: the Sirt1-deficient group displayed a mild pro-calcific trend, while the Sirt1-overexpressing group showed partial protection, though neither reached statistical significance.

Next, we investigated whether SIRT1 deficiency could reprogram VICs towards an osteogenic phenotype. SIRT1 silencing and hyperphosphate medium treatment exacerbated hyperphosphate medium-induced increase in the levels calcium nodule formation (Fig. 3i, l), ALP activity (Fig. 3j, m), as well as β-galactosidase (β-gal) staining (Fig. 3k, n), while up-regulation of SIRT1 harvested opposite results. Taken together, these results indicated that SIRT1 deficiency accelerated CKD-induced aortic valve calcification and senescence both in vivo and in vitro.

### NLRP3 inflammasome mediated the effects of SIRT1 deficiency on CKD-induced aortic valve calcification

**Fig. 4:**
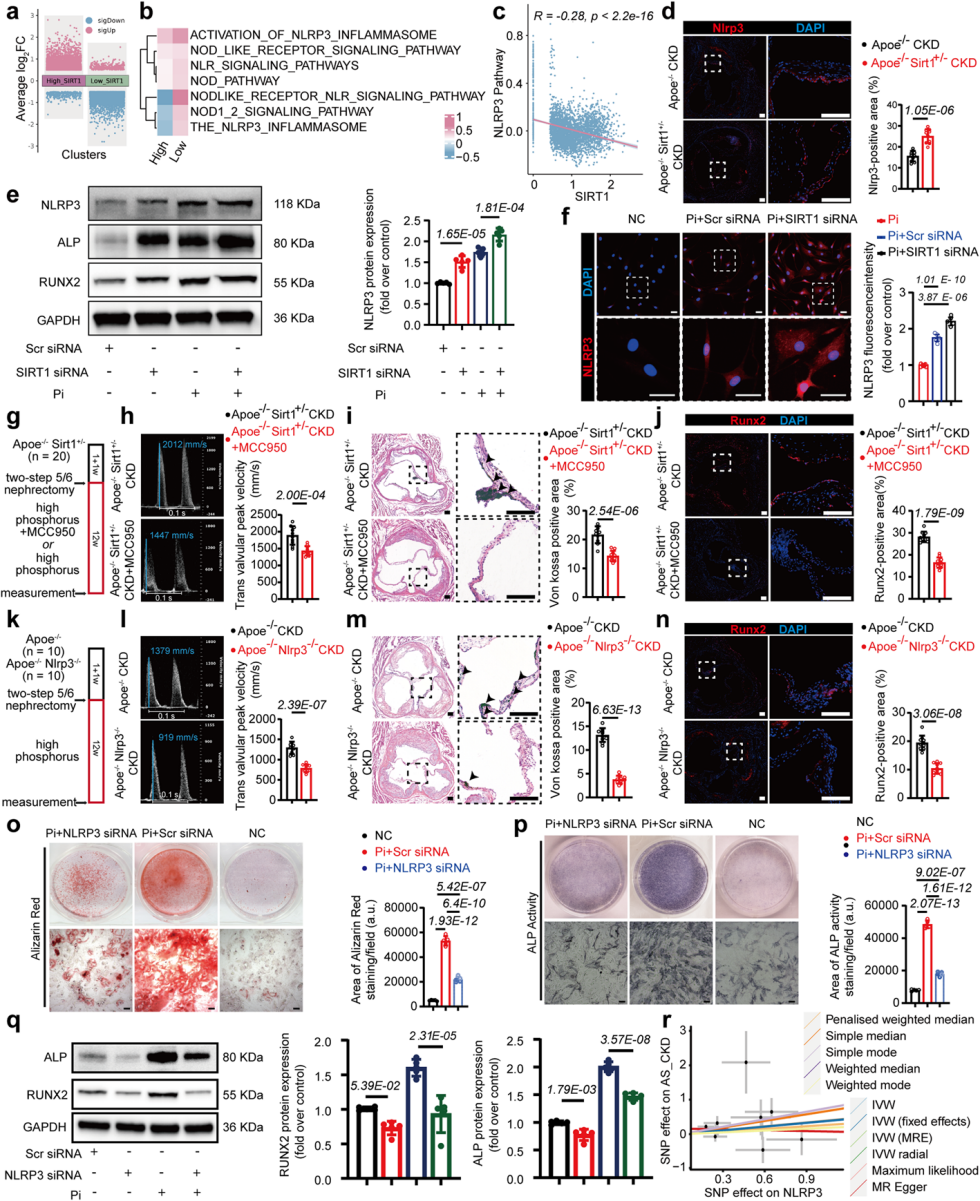
NLRP3 inflammasome mediated the effects of SIRT1 deficiency on CKD-induced aortic valve calcification. **a** Differential gene volcano plots of high SIRT1-expressing subpopulations versus low SIRT1-expressing subpopulations in myofibroblasts from the aforementioned single-cell dataset. **b** GSVA pathway enrichment analysis revealed that NOD-like pathways were significantly upregulated in the SIRT1 low-expression subpopulation. **c** Scatter plots showed a significant negative correlation between SIRT1 expression and NLRP3 pathway score. **d** Representative images of immunofluorescence staining and quantification analysis showed that *Sirt1*^*+/−*^ *Apoe*^*−/−*^ CKD mice had significantly higher expression of Nlrp3 (red) than *Apoe*^*−/−*^ CKD mice. DAPI (blue) was used for nuclear counterstaining. *n* = 10 per group. Scale bar = 100 μm. e Representative western blot bands showed ALP, RUNX2, and NLRP3 expression in VICs treated with hyperphosphate culture medium for 72 h after SIRT1 siRNA transfection; densitometric quantification is shown for NLRP3. *n* = 5 per group. **f** Representative images of immunofluorescence staining and quantification analysis showed that silencing SIRT1 VICs had significantly higher expression of NLRP3 (red), DAPI (blue) was used for nuclear counterstaining. *n* = 5 per group. Scale bar = 25 μm. **g** Schematic diagram of establishing and monitoring Sirt1 and Nlrp3 co-intervention CKD induced CAVD mice model. **h** Echocardiography showed that *Sirt1*^*+/−*^ *Apoe*^*−/−*^ + MCC950 CKD mice had a significantly reduced transvalvular peak jet velocity than *Sirt1*^*+/−*^*Apoe*^*−/−*^ CKD mice. *n* = 10 per group. **i** Representative images of von Kossa staining of calcium in aortic valves of CKD mice. *n* = 10 per group. Calcium deposition was significantly decreased in aortic valves of *Sirt1*^*+/−*^ *Apoe*^*−/−*^ + MCC950 CKD mice compared with *Sirt1*^*+/−*^*Apoe*^*−/−*^ CKD mice. *n* = 10 per group. Scale bar = 100 μm. **j** Representative images of immunofluorescence staining and quantification analysis showed that *Sirt1*^*+/−*^ *Apoe*^*−/−*^ + MCC950 CKD had significantly lower expression of Runx2 (red) in aortic valves than *Sirt1*^*+/−*^ *Apoe*^*−/−*^ CKD mice. DAPI (blue) was used for nuclear counterstaining. *n* = 10 per group. Scale bar = 100 μm. **k** Schematic diagram of establishing and monitoring Nlrp3 knockout CKD induced CAVD mice model. *n* = 10 per group. **l** Echocardiography showed that *Nlrp3*^*−/−*^*Apoe*^*−/−*^ CKD mice had a significantly reduced transvalvular peak jet velocity than *Apoe*^*−/−*^ CKD mice. *n* = 10 per group. **m** Representative images of von Kossa staining of calcium in aortic valves of CKD mice. *n* = 10 per group. Calcium deposition was significantly decreased in aortic valves of *Nlrp3*^*−/−*^*Apoe*^*−/−*^ CKD mice compared with *Apoe*^*−/−*^ CKD mice. *n* = 10 per group. Scale bar = 100 μm. **n** Representative images of immunofluorescence staining and quantification analysis showed that *Nlrp3*^*−/−*^*Apoe*^*−/−*^ CKD mice had significantly lower expression of Runx2 (red) in aortic valves than *Apoe*^*−/−*^ CKD mice. DAPI (blue) was used for nuclear counterstaining. *n* = 10 per group. Scale bar = 100 μm. **o** and **p** Representative images of staining and quantification analysis showed down-regulating NLRP3 led to reducing of alizarin red level and ALP activity. *n* = 5 per group. Scale bar = 25 μm. **q** Representative western blot band and densitometric quantification showed that NLRP3 siRNA significantly decreased ALP and RUNX2 expression in VICs treated with hyperphosphate culture medium for 72 h. *n* = 5 per group. **r** Scatterplot of expression quantitative trait loci (eQTL) Mendelian randomization (MR) analysis on NLRP3 expression levels and the occurrence of AS in CKD patients

To elucidate the downstream inflammatory mechanisms linking SIRT1 deficiency to valve calcification, we focused on the NLRP3 inflammasome pathway. Although a positive correlation between SIRT1 deficiency and inflammation was partially observed in the ELISA experiments described above, the exact followed mechanism was still unclear. Myofibroblast-VICs subgroups derived from the aforementioned scRNA sequencing results were divided into two populations based on median SIRT1 expression (Fig. 4a). Differential gene and pathway enrichment was performed. The results showed that the subpopulation of myofibroblasts with low expression of SIRT1 had widespread activation of NOD-like pathways, with the NLRP3 pathway being the most widely upregulated (Fig. 4b). Correlation analysis showed that the level of SIRT1 exhibited a significant negative correlation with the NLRP3 pathway (Fig. 4c). Immunofluorescence staining displayed increased expression of NLRP3 in aortic valves of *Sirt1*^*+/−*^ *Apoe*^*−/−*^ mice compared with that in *Apoe*^*−/−*^ mice (Fig. 4d). Western blot analysis of VICs exhibited an increase in NLRP3 after intervention of SIRT1 (Fig. 4e). In vitro immunofluorescence staining confirmed the increased expression after SIRT1 inhibition in VICs from aortic valves (Fig. 4f). However, rescue treatment with resveratrol or BAY11-7082 significantly corrected the upregulation of NLRP3 induced by hyperphosphate medium (Fig. S12a-12f).

To further investigate the mediating effect of NLRP3 between SIRT1 and valve calcification, MCC950 (10 mg/kg) was given every week through tail vein injection (Fig. 4g). After 12 weeks of MCC950 treatment to *Sirt1*^*+/−*^ *Apoe*^*−/−*^ CKD mice, we found that transvalvular peak jet velocity of aortic valve was significantly decreased in the MCC950-injection group compared to control group (Fig. 4h). Von Kossa staining also showed that MCC950-injection group represented a smaller valve calcification area than control group (Fig. 4i). Calcium detection by Alizarin Red S staining showed similar patterns to those observed with von Kossa staining (Fig. S13). Furthermore, immunofluorescence staining showed decreased Runx2 expression in aortic valves of *Sirt1*^*+/−*^ *Apoe*^*−/−*^ mice with MCC950 treatment (Fig. 4j).

To confirm whether the activation of NLRP3 inflammasome is involved in CKD-induced aortic valve calcification, we generated CKD condition in *Apoe*^*−/−*^ mice (*n* = 10) and *Nlrp3*^*−/−*^*Apoe*^*−/−*^ mice (*n* = 10), and fed them with high-phosphate fodder for 12 weeks (Fig. 4k). After 12 weeks of high-phosphate feeding, transvalvular peak jet velocity of aortic valve was significantly lower in *Nlrp3*^*−/−*^*Apoe*^*−/−*^ mice compared with *Apoe*^*−/−*^ mice (Fig. 4l). Von Kossa staining and immunofluorescence staining of Runx2 showed *Nlrp3*^*−/−*^*Apoe*^*−/−*^ mice exhibited smaller valve calcification area, decreased Runx2 expression compared with *Apoe*^*−/−*^ mice (Fig. 4m-n). Alizarin Red S staining yielded consistent results. After 21 days of cell culture, Alizarin Red staining showed hyperphosphate medium could trigger notable calcium nodules formation, while NLRP3 siRNA significantly attenuated this effect (Fig. 4o), as was the same for ALP activity (Fig. 4p). Moreover, we further investigated the role of NLRP3 in vitro, VICs were transfected with scrambled siRNA and NLRP3 siRNA accordingly. Western blot analysis showed that ALP and RUNX2 expression was suppressed when VICs were transfected with NLRP3 siRNA in the presence or absence of high-phosphate (Fig. 4q). In summary, these results demonstrated NLRP3 inflammasome activation leading to the deterioration of CKD-induced aortic valve calcification.

Genetic variations in the NLRP3 gene are associated with the risk of AS in the same large-scale cohort. eQTL-MR demonstrated that NLRP3 expression in valve-related tissue positively correlated to odds ratio of aortic stenosis occurrence, significant in maximum likelihood, MR Egger, penalized weighted median and IVW fixed effects method (Fig. 4r, Table S4). In summary, SIRT1 deficiency aggravated CKD-induced aortic valve calcification through activation of NLRP3 inflammasome.

### SIRT1 inhibited high phosphorous-associated NLRP3 inflammasome activation by suppressing the glycolysis-induced NF-κB pathway

**Fig. 5:**
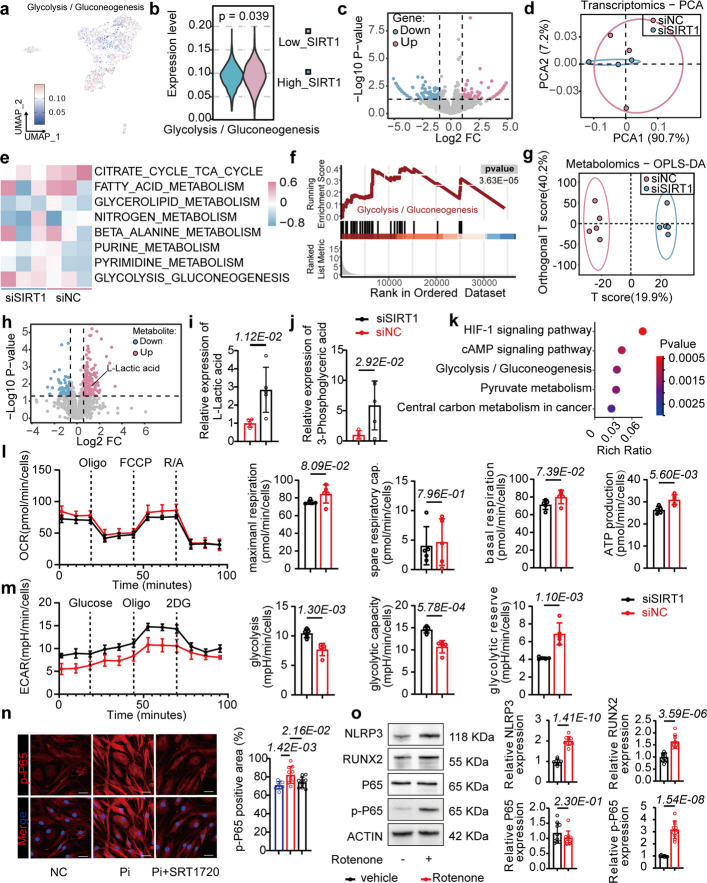
SIRT1 suppressed high phosphorous-associated NF-κB-NLRP3 pathway activation by inhibiting glycolysis. **a** Reduction plot of glycolysis pathway score in myofibroblast-VICs. **b** Violin plot of glycolysis pathway in myofibroblast-VICs. **c** Volcano plot comparing gene expression between siNC/siSIRT1-treated VICs (*n* = 3). **d** Principal component analysis (PCA) revealing inter-sample (siNC and siSIRT1) agreement. **e** Heatmap comparing the enrichment score of key metabolic pathways; analysis of RNA-seq expression (*n* = 3). **f** GSEA reveals that glycolysis/glyconeogenesis pathway was enriched in siSIRT1 group. **g** Orthogonal Partial Least Squares Discriminant Analysis (OPLS-DA) plot of the metabolomic profiles. **h** Volcano plot of untargeted metabolomic profiling of siNC/siSIRT1-treated VICs (*n* = 5). **i** Relative expression of L-Lactic acid (*n* = 5). **j** Relative expression of 3-Phosphoglyceric acid (*n* = 5). **k** The enrichment analysis of the metabolomic data. **l-m** Real-time changes in the OCR (**l**) and ECAR (**m**) of VICs, pretreated with siSIRT1 or siNC, measured using Seahorse. Oligo, oligomycin; FCCP, carbonyl cyanide4-(trifluoromethoxy) phenylhydrazone; R/A, rotenone plus antimycin A; 2-DG, 2-deoxy-d-glucose. Basal respiration, spare respiratory capacity, maximal respiration, and ATP production were determined based on OCR readings. Glycolytic reserve, glycolysis, and glycolytic capacity were extracted from ECAR. (*n* = 5). **n** Immunofluorescence staining of p-P65 (Red) and DAPI (Blue). *n* = 5 per group. Quantification of the percentage of the p-P65 positive area. *n* = 5 per group. Scale bar = 25 μm. **o** Representative western blot images of NLRP3, RUNX2, P65, p-P65 and ACTIN of VICs treated with Rotenone (10 nM, 48 h) or control. *n* = 5 per group. Quantification of western blot analysis for NLRP3, RUNX2, P65, p-P65. *n* = 5 per group

To further dissect the metabolic and signaling mechanisms downstream of SIRT1, we investigated its role in regulating cellular metabolism and inflammatory signaling pathways in VICs. Comparison between high and low SIRT1 expression myofibroblast-VICs indicated an up-regulation of glycolysis (Fig. 5a, b, k). To further elucidate the precise mechanism through which SIRT1 modulated the inflammatory response, we undertook a comprehensive analysis of the transcriptomic and metabolomic profiles. By comparing the transcriptomic data from siSIRT1 to siNC, we were able to identify a total of 345 differentially expressed genes (DEGs), of which 169 were up-regulated and 176 were down-regulated (Fig. 5c, d). The DEGs were found to be enriched in metabolic pathways, including TCA cycle, fatty acid metabolism, purine metabolism and glycolysis (Fig. 5e), among which, the glycolysis pathway demonstrated significant enrichment (Fig. 5f). The overall expression profile of the non-targeted metabolome demonstrated a notable distinction between the siSIRT1 and siNC groups (Fig. 5g, h). A total of 210 differential metabolites were obtained, of which 147 were up-regulated and 63 were down-regulated (Fig. 5h, Fig. S14). It is noteworthy that the end product of glycolysis, L-Lactic acid, demonstrated a significant up-regulation (Fig. 5i). Similarly, one of the intermediate products of glycolysis, 3-Phosphoglyceric acid, also exhibited a significant up-regulation (Fig. 5j). The differential metabolites were significantly enriched in signaling pathways such as HIF-1 glycolysis pathway and pyruvate metabolism pathway (Fig. 5k). Based on these findings, it can be proposed that SIRT1 negatively regulates the glycolytic pathway.

We then investigated the effects of SIRT1 on mitochondrial oxidative phosphorylation and glycolysis by monitoring the oxygen consumption or extracellular acidification rate (OCR or ECAR, respectively) of VICs after knockdown of SIRT1 or administration of SIRT1 agonists (Fig. 5l, m). Our results showed that siSIRT1 significantly inhibited maximal respiratory capacity and ATP synthesis (Fig. 5l), while enhancing the dependence of VIC on glycolysis (Fig. 5m). In addition, we found that the SIRT1 agonist SRT1720 inhibited glycolytic processes while promoting mitochondrial oxidative phosphorylation (Fig. S15). Hypoxia and glycolysis have long been thought to promote activation of the immune response [18]. We hypothesized that SIRT1 may modulate the inflammatory response in a similar way. Phosphorylation of P65 was significantly reduced after administration of SRT1720 (Fig. 5n). Similar results were received in p/t-p65 and p/t-IKBα (Fig. S16). In contrast, after endogenous blockade of oxidative phosphorylation by administration of rotenone, phosphorylation of P65 was significantly enhanced and accompanied by significant upregulation of RUNX2 and NLRP3 (Fig. 5o). In summary, SIRT1 may inhibit high phosphorous-associated NLRP3 activation by suppressing the glycolysis-induced NF-κB pathway.

### Anti-diabetic compound screening revealed semaglutide attenuated CKD-induced aortic valve calcification

**Fig. 6:**
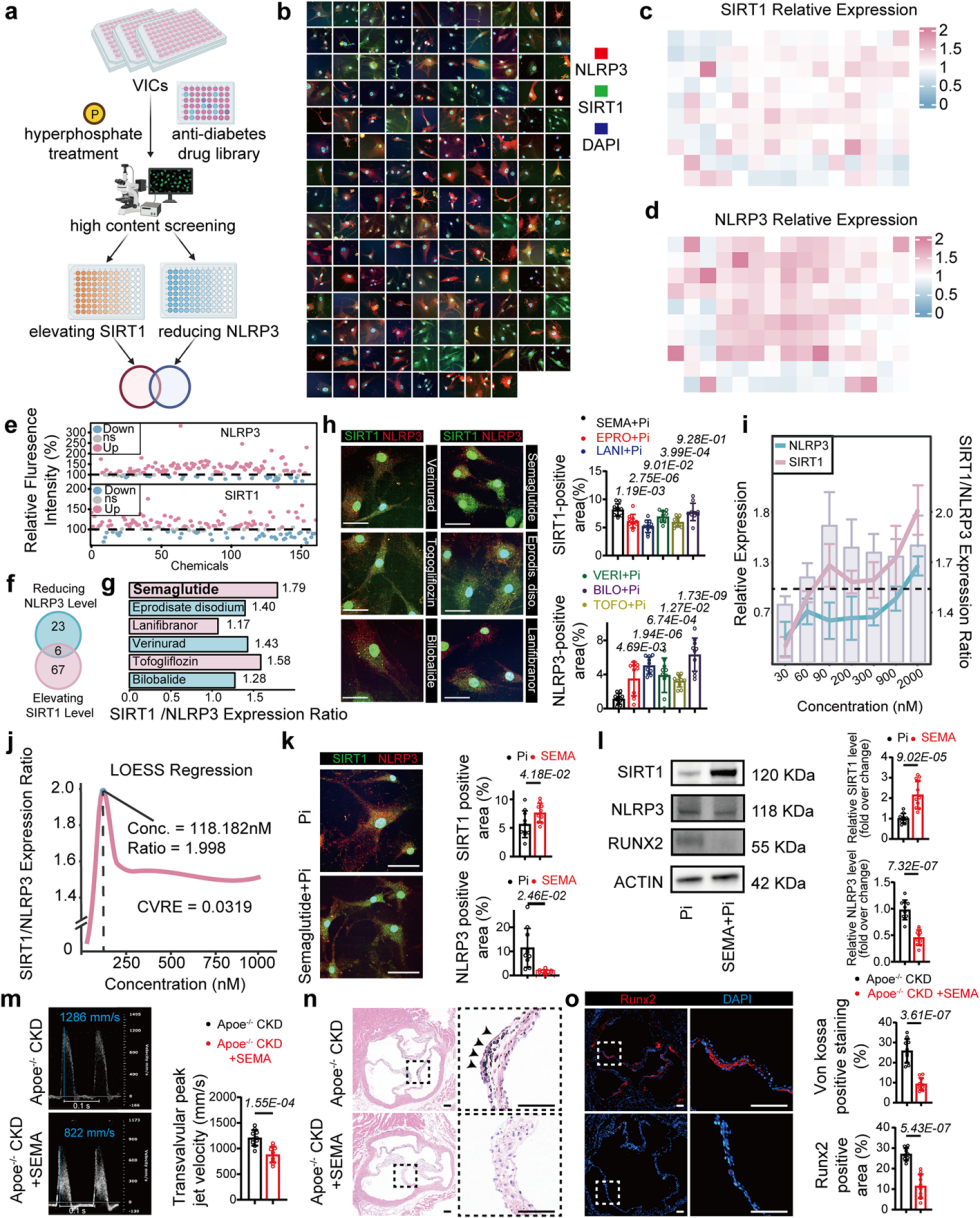
Anti-diabetic compound screening revealed semaglutide attenuated CKD-induced aortic valve calcification. **a** Schematic diagram of high content anti-diabetic compound screening on VICs. **b** Representative images of immunofluorescence staining screening after different compound treatment. NLRP3 (red), SIRT1 (green), and DAPI (blue) was used for nuclear counterstaining. *n* = 3 per group. **c** Representative heatmap of SIRT1 expression after different compound treatment. **d** Representative heatmap of NLRP3 expression after different compound treatment. **e** Scatterplot showed the statistical level change of SIRT1 and NLRP3 after treatment. **f** Venn diagram indicated 6 compounds were candidate for CAVD treatment. **g** Statistical level change of SIRT1/NLRP3 ratio of 6 compounds after treatment. *n* = 5 per group. **h** Representative images of immunofluorescence staining and quantification analysis showed that semaglutide had significantly lowered NLRP3 (red) and elevated SIRT1 (green) level in VICs. DAPI (blue) was used for nuclear counterstaining. *n* = 5 per group. Scale bar = 25 μm. **i** SIRT1/NLRP3 ratio and separate levels after semaglutide treatment on VICs under a sequenced concentration gradient. **j** LOESS regression demonstrated 118.182 nM would be the most fitting concentration. **k** Representative images of immunofluorescence staining and quantification analysis showed that semaglutide treatment under the best concentration had significantly lowered NLRP3 (red) and elevated SIRT1 (green) level in VICs. *n* = 5 per group. Scale bar = 25 μm. **l** Representative western blot images of NLRP3, SIRT1 and RUNX2 of VICs treated with semaglutide (118.182 nM, 72 h). Quantification of western blot analysis for NLRP3 and SIRT1. **m** Echocardiography showed that semaglutide treated *Apoe*^*−/−*^ CKD mice had a significantly reduced transvalvular peak jet velocity than *Apoe*^*−/−*^ CKD mice. *n* = 10 per group. **n** Representative images of von Kossa staining of calcium in aortic valves of CKD mice. Calcium deposition was significantly decreased in aortic valves of semaglutide treated *Apoe*^*−/−*^ CKD mice compared with *Apoe*^*−/−*^ CKD mice. *n* = 10 per group. Scale bar = 100 μm. **o** Representative images of immunofluorescence staining and quantification analysis showed that semaglutide treated *Apoe*^*−/−*^ CKD mice had significantly lower expression of Runx2 (red) in aortic valves than *Apoe*^*−/−*^ CKD mice. DAPI (blue) was used for nuclear counterstaining. *n* = 10 per group. Scale bar = 100 μm. Created with BioRender.com

To explore potential therapeutic strategies targeting the SIRT1-NLRP3 axis, we performed high-throughput screening of anti-diabetic compounds in VICs. Since pathological VICs present significant abnormalities in glucose metabolism, coupled with the immense potential of anti-diabetic drugs shown recently for controlling the development of cardiovascular disease, we introduced 148 anti-diabetic compounds in the Selleck drug library for screening in VICs (Fig. 6a, Table S5). High content cell imaging system evaluated the change of SIRT1 and NLRP3 expression after compound treatment (Fig. 6b-d). Among 148 compounds, 29 posed a potential role to reduce NLRP3 expression level and 73 posed potentials to elevate SIRT1 expression level (Fig. 6e, f). Six compounds, semaglutide, eprodisate disodium, lanifibranor, verinurad, tofogliflozin, and bilobalide showed capability of both reducing NLRP3 expression level and elevating SIRT1 expression level (Fig. 6g). To determine the best compound treating CKD induced CAVD, we repeated screening in six compounds individually under default concentration of 10 nM. Screening results echoed with high content screening results, and semaglutide topped the effects of SIRT1 elevating and NLRP3 reducing among six compounds (Fig. 6h-i).

Next, we evaluated the SIRT1/NLRP3 ratio of semaglutide on VICs under a sequenced concentration gradient, and applied LOESS regression to obtain the best concentration. Regression outcome indicated 118.182 nM to be the best concentration, under which the SIRT1/NLRP3 ratio would reach 1.988 (CVRE = 0.0319) (Fig. 6j). Immunofluorescence staining of VICs under the best concentration showed semaglutide significantly lowered the NLRP3 expression level and elevated the SIRT1 expression level (Fig. 6k). Western blot analysis also exhibited that semaglutide’s capability of elevating SIRT1/NLRP3 expression level and lowering RUNX2 level, showed its potential for alleviating the development of CAVD (Fig. 6l).

Prior to in vivo experiments, we conducted dose exploration to evaluate the efficacy and tolerability of semaglutide across a range of doses (Fig. S17a). The results indicated a dose-dependent protective trend on aortic valve calcification and related functional parameters. Among the tested doses, 60 μg/kg/day (subcutaneous injection daily for 4 consecutive weeks) achieved the best overall efficacy. Evaluation of plasma biochemical indicators showed no significant adverse changes at this dose, suggesting favorable safety and tolerability (Fig. S18). In vivo experiments showed that *Apoe*^*−/−*^ CKD mice treated with semaglutide had a significantly reduced transvalvular peak jet velocity, calcium deposition and expression of Runx2 in aortic valves compared to *Apoe*^*−/−*^ CKD mice (Fig. 6m-o). To further determine whether the protective effects of semaglutide are SIRT1-dependent, we performed SIRT1 loss-of-function experiments both in vitro and in vivo. In vitro, SIRT1 knockdown via siRNA markedly attenuated the inhibitory effects of semaglutide on osteogenic markers and calcification in VICs (Fig. S17b). In vivo, semaglutide treatment in *Sirt1*^*mfKO*^* Apoe*^*−/−*^ CKD mice failed to confer protection against aortic valve calcification, as evidenced by unchanged transvalvular flow velocity and persistent Alizarin Red S staining of calcium deposition (Fig. S17c-17d). Taken together, these findings identify semaglutide as a promising therapeutic candidate that modulates the SIRT1-NLRP3 axis to attenuate CKD-induced aortic valve calcification.

## Discussion

CKD is a major risk factor for aortic valve calcification, yet the mechanisms linking renal dysfunction to valvular pathology remain incompletely defined. Integrating single-cell transcriptomics, human genetics, and functional studies, we identify SIRT1 as a central regulator connecting metabolic dysregulation to inflammatory activation in CKD-associated valve calcification. SIRT1 deficiency drives coordinated metabolic-inflammatory reprogramming in VICs, promoting glycolysis, NF-κB and NLRP3 activation, and ultimately osteogenic differentiation and calcification. This metabolic-inflammatory coupling links CKD-associated inflammaging to valvular disease progression. Genetic and pharmacological evidence, including semaglutide intervention, further supports the functional relevance of the SIRT1-NLRP3 axis as a potential therapeutic target.

CAVD is a common and frequently occurring disease in the elderly, leading to aortic stenosis and resulting in subsequent responses like cardiac hypertrophy and heart failure [5, 19] CKD greatly accelerates the progression of aortic valve calcification, with 10–20 years earlier in CKD patients than general population [20]. Previous studies have suggested that the advance of CKD is often accompanied by the development of vascular calcification and the ageing process [21]. Both CKD and aging are among the principal risk factors for CAVD. However, unlike the discoveries of pathogenesis of other cardiovascular diseases such as atherosclerosis and vascular calcification, the specific mechanism of valve calcification induced by CKD has not been fully investigated yet.

SIRT1, a human orthologue of yeast Sir2, regulates multiple intracellular processes via deacetylation of histone and non-histone lysine residues [22]. Its protective role has been well studied in cardiovascular diseases including atherosclerosis, vascular calcification, cardiomyopathy, and heart failure [23]. Since aortic valve calcification is aging-related and SIRT1 is crucial for cellular senescence, it may link CKD to valve calcification. SIRT1 is downregulated in calcified aortic valves in CKD patients, partly due to phosphorus metabolism disorders, and this deficiency may promote VIC senescence, pro-inflammatory activation, and osteogenic differentiation. Beyond its classical anti-inflammatory effects, SIRT1 regulates glucose and lipid metabolism and influences cellular states and cardiovascular protection by maintaining energy homeostasis [24]. It modulates the balance between glycolysis and fatty acid oxidation, affecting inflammation and cellular function [25]. In kidney disease, SIRT1 promotes lipid metabolism, suppresses glycolysis, and mitigates lipotoxicity, suggesting its metabolic regulation complements anti-inflammatory mechanisms [26]. In CKD-associated CAVD, SIRT1 deficiency may thus drive VICs into a pathological state with metabolic dysregulation, heightened inflammation, and increased osteogenic potential, promoting valve calcification.

Older organisms tend to develop a pro-inflammatory status, termed inflammageing, characterized by elevated circulating cytokines such as CRP, IL-1β, and IL-18 [27], which contributes to CKD and cardiovascular diseases [28]. As an anti-aging regulator, SIRT1 also modulates inflammatory responses [29]. Overexpression of SIRT1 deacetylates RELA/p65 of NF-κB, restraining its activity and downstream cytokine secretion [13]. In our study, NLRP3, a downstream target of NF-κB, was upregulated in SIRT1-deficient *Apoe*^*−/−*^ CKD mice. NLRP3 inflammasome activation produces pro-inflammatory cytokines IL-1β and IL-18. Liu et al. [30] showed that NF-κB/NLRP3/IL-1 signaling promotes osteoblastic differentiation and VIC calcification [30], which our data confirmed and extended: MCC950, a specific NLRP3 inhibitor, attenuated valve calcification in *Sirt1*^*+/−*^ *Apoe*^*−/−*^ CKD mice, and NLRP3 knockout delayed calcification in CKD *Apoe*^*−/−*^ mice. In vitro, SIRT1 deficiency increased IκBα phosphorylation, promoting NF-κB nuclear translocation and subsequent NLRP3 activation. These results suggest that SIRT1 protects against CKD-induced aortic valve calcification by inhibiting NLRP3 and downstream inflammation.

Metabolism and immune response are tightly linked [31]. Activated myeloid cells shift from oxidative phosphorylation to glycolysis to support immune function [31, 32]. SIRT1 acts as a metabolic sensor, regulating this transition and the inflammatory response [33]. It inhibits glycolytic enzymes via β-catenin deacetylation [33], whereas glycolytic metabolite accumulation from SIRT1 downregulation can activate inflammation via mTOR or histone modifications [34]. In VICs, SIRT1 may control inflammation by modulating glycolysis, highlighting how valvular calcification occurs in a hypoxic microenvironment with metabolic shifts, though further mechanistic studies are needed.

Decreased endothelial SIRT1 impairs eNOS activity and NO bioavailability, contributing to early valvular dysfunction. Endothelium-derived NO protects aortic valve homeostasis, whereas impaired NO promotes VIC osteogenic differentiation and matrix calcification [35]. CKD-associated stress may further weaken endothelial SIRT1, suppressing eNOS/NO signaling, exacerbating dysfunction, and creating a pro-calcific microenvironment [36, 37]. Intrinsic VIC SIRT1 also plays a critical role, with its downregulation linked to enhanced calcification, inflammation, and osteogenic differentiation, indirectly amplifying valve calcification [38, 39]. Thus, VEC and VIC mechanisms likely interact, with the SIRT1-eNOS-NO axis explaining early endothelial injury and the SIRT1-NF-κB-NLRP3 pathway in VICs mediating sustained inflammation and pathological calcification.

Drug screening suggested semaglutide may have therapeutic potential. Two international, multicenter, double-blind, placebo-controlled trials indicate semaglutide benefits cardiovascular health in obesity-associated HFpEF beyond weight loss [40]. Besides glycemic regulation, it shows anti-inflammatory effects; in a pentylenetetrazole-induced epilepsy model, it inhibited NLRP3-mediated cytokine release [41, 42]. Since inflammation is central to CAVD, semaglutide may be therapeutically relevant. Currently, valve repair or replacement is the only proven CAVS treatment, and no drugs prevent or slow CAVD [43, 44]. Traditional cardiovascular agents fail to delay calcification, highlighting interest in GLP-1RAs and SGLT2 inhibitors for their cardiometabolic benefits [45]. Semaglutide reduces MACE, mortality, and non-fatal MI, improving outcomes via blood pressure reduction, weight loss, glycemic control, and oxidative stress mitigation [46–48]. Mechanistically, it may activate AMPK/SIRT1 to improve oxidative stress and regulate autophagy and mitochondrial dynamics [49]. Other GLP-1RAs, like liraglutide, inhibit aortic valve calcification [50], and endogenous GLP-1 dose-dependently suppresses VIC calcification and osteogenic gene expression in vitro [51]. Further animal studies and clinical trials are needed.

VECs play a key role in early CAVD initiation. Endothelial SIRT1 enhances eNOS activity, suppresses inflammation, mitigates ER stress, and delays senescence, providing vascular protection and anti-calcification effects [52, 53]. In CKD, uremic toxins, inflammation, and oxidative stress impair endothelial function, disrupt integrity, upregulate pro-inflammatory factors, and create a microenvironment that may trigger early CAVD via paracrine signaling [54]. SIRT1 decline in CKD is linked to vascular calcification, while its activation can reduce calcification [55]. We focus on VICs because they are the principal effectors of aortic valve calcification, controlling lesion formation through matrix remodeling, inflammatory signaling, and osteogenic differentiation [56, 57]; scRNA-seq shows SIRT1 enrichment in VIC subpopulations, especially in CKD-associated CAVD valves. VEC-VIC interactions are dynamic: VECs can undergo EndMT and influence VIC activation, while VICs reciprocally regulate VEC EndMT and osteogenic differentiation via paracrine signaling, with disrupted interactions exacerbating calcification [58, 59]. VECs modulate VIC differentiation through NO signaling, oxidative stress, and EndMT pathways, highlighting their role in shaping VIC activation [60].

Consistent with previous studies, our results confirm SIRT1’s protective role in suppressing calcification. Spermidine attenuates CKD-associated vascular calcification via SIRT1 upregulation and ER stress inhibition, whereas SIRT1 inhibition abolishes this effect [61]. Reviews also link SIRT1 downregulation in CKD to vascular calcification, suggesting therapeutic potential [55]. Similarly, we observed marked SIRT1 downregulation in CKD patient and mouse aortic valves, accompanied by enhanced calcification and senescence. Unlike prior studies focusing on VSMCs, scRNA-seq revealed that SIRT1 downregulation mainly occurs in myofibroblast-VICs, enriched for senescence and osteogenic pathways, with trajectory analysis showing a shift toward osteogenic differentiation, a novel CKD-specific finding. In high-phosphate CKD conditions, SIRT1 deficiency promotes osteogenesis and metabolic reprogramming, activating NF-κB/NLRP3 inflammasomes [53, 62, 63]. Building on this, our study expands the SIRT1 regulatory network. Integrative transcriptomic and metabolomic analyses showed that SIRT1 loss decreases oxidative phosphorylation, enhances glycolysis, and drives VIC osteogenic differentiation. Multi-level evidence, including UK Biobank cohort analysis, eQTL-MR, SIRT1 gene-modified mice, and NLRP3/MCC950 interventions, strengthens causal inference. Despite limited options for CKD-related CAVD and the ineffectiveness of conventional cardiovascular drugs [8], high-throughput screening identified semaglutide as elevating the SIRT1/NLRP3 ratio and alleviating valve calcification in CKD mice, potentially via the AMPK/SIRT1 axis and modulation of oxidative stress, autophagy, and mitochondrial dynamics [49]. This study is the first to link GLP-1RAs to CKD-associated CAVD through SIRT1-NLRP3, proposing restoration of SIRT1-mediated metabolic-inflammatory balance as a potential therapeutic strategy.

Several limitations of this study should be acknowledged. First, although we integrated large-scale population data from the UK Biobank with genetic analyses to support a potential causal relationship, the observational nature of these data cannot fully exclude residual confounding. Second, the single-cell RNA sequencing analysis was performed on a limited number of human valve samples, which may not fully capture the heterogeneity of CKD-associated valvular calcification. Third, while our in vivo and in vitro experiments provide mechanistic insights into the SIRT1-NF-κB-NLRP3 axis, the complexity of CKD-related metabolic and inflammatory alterations suggests that additional pathways may also contribute to disease progression. Fourth, although semaglutide demonstrated therapeutic potential in experimental models, its efficacy and safety in patients with CKD-associated aortic valve calcification require further validation in prospective clinical studies. Finally, the temporal and cell-type-specific roles of SIRT1, particularly in valve endothelial cells versus interstitial cells, remain to be further elucidated.

In conclusion, the novel findings of the present study suggest that SIRT1 deficiency may promote CKD-induced aortic valve calcification, which may subsequently lead to NF-κB and NLRP3 activation, and thus osteoblastic differentiation and calcium nodule formation of VICs. Semaglutide supplementation could help to mitigate hyperphosphatemia-induced aortic valve calcification in CKD mice by salvaging SIRT1 bioavailability. Semaglutide may represent a potential preventive strategy for aortic valve calcification in CKD patients.

## Materials and methods

### Cell culture and treatment

Primary valve interstitial cells (VICs) were isolated from human aortic valve tissues obtained from patients undergoing heart transplantation or valve replacement, as described previously [64]. Cells were cultured in high-glucose DMEM supplemented with 10% fetal bovine serum, 100 U/mL penicillin, and 100 μg/mL streptomycin at 37 °C with 5% CO₂. For osteogenic differentiation, VICs were cultured under high-phosphate osteogenic conditions (DMEM + 10% FBS + 100 U/mL penicillin + 100 μg/mL streptomycin + 10 mmol/L β-glycerophosphate and 3 mmol/L CaCl₂) for up to 21 days, with medium refreshed every 2–3 days, representing a high-phosphate-driven osteogenic/calcification model. This model was used to evaluate time-dependent osteogenic differentiation and mineralization. Pharmacological interventions were performed using SRT1720 (10 μM) and MCC950 (100 nM) where indicated. Detailed procedures are provided in the Supplementary Methods.

### Human aortic valve samples collection

Human aortic valve samples were collected from patients undergoing cardiac surgery. Calcific aortic valve tissues were obtained from patients undergoing aortic valve replacement with a diagnosis of calcific aortic stenosis and CKD, whereas control aortic valve tissues were collected from patients undergoing heart transplantation due to dilated cardiomyopathy. Patients with rheumatic valve disease, bicuspid aortic valve, infective endocarditis, or isolated aortic regurgitation were excluded. A total of 40 human aortic valve samples were included in this study, comprising 20 CAVD samples and 20 control samples. Detailed sample metadata is provided in Table S2 and S3.

Fresh aortic valve tissues were processed immediately after surgery according to downstream experimental requirements. For single-cell RNA sequencing, valve tissues were washed with cold 1 × PBS and mechanically dissociated using fine scissors. Tissue fragments were then digested in DMEM containing collagenase type I (2 mg/mL; Sigma-Aldrich, Saint Louis, MO, USA) to generate single-cell suspensions. The resulting cell suspensions were transported on ice to the BGI Wuhan Research Center for single-cell sequencing, and cell viability was maintained at ≥ 90%. Additional collected tissues were processed for protein extraction, immunofluorescence staining, and primary cell isolation as described in the Supplementary Methods.

### Single-cell RNA sequencing

Single-cell suspensions were prepared from freshly isolated aortic valve tissues by enzymatic digestion using collagenase I. Single-cell gene expression libraries were prepared following the Chromium Single Cell 3′ v2 protocol (10 × Genomics; Documents CG00052 and CG00055). After reverse transcription and emulsion breakage, libraries were constructed according to the manufacturer’s instructions and sequenced on an Illumina HiSeq X Ten system.

Raw sequencing reads were processed using CellRanger (v3.0.2) and aligned to the human reference genome (hg19) via STAR (v2.5.1b43). Quality control excluded cells expressing fewer than 200 genes or with > 50% mitochondrial gene content. Doublets were removed using DoubleFinder (v2.0.3). Data normalization, scaling, and identification of highly variable genes were performed using Seurat (v4.2.0). Data integration across samples used FindIntegrationAnchors with 2000 genes per sample, and batch effects were corrected using the Harmony R package (v0.1.1). Dimensionality reduction was performed using UMAP, and clustering was conducted in Seurat. Differentially expressed genes (DEGs) were identified using the Wilcoxon rank-sum test (FindAllMarkers), with adjusted *P* < 0.05 considered significant. Pathway enrichment analysis was conducted using fgsea (v1.27.1) with gene sets from msigdb (v7.5.1).

Trajectory analyses were performed using Monocle3 (v1.3.4) and Slingshot (v2.1.0.0) based on minimum spanning tree algorithms, with starting points defined according to DEG profiles and biological relevance. Plot visualization and refinement were performed using SCP (v0.5.3) and plot1cell (v0.0.0.9000).

### Experimental animals

All animal procedures conformed to the current NIH Guide for the Care and Use of Laboratory Animals and were approved by the Institutional Animal Research Committee of Tongji Medical College, Huazhong University of Science and Technology. Eight-week-old male *Apoe*^*−/−*^ mice, *Sirt1*^*+/−*^ *Apoe*^*−/−*^ mice and *Nlrp3*^*−/−*^*Apoe*^*−/−*^ mice on C57BL/6 background were purchased from Gene&Peace biotech Co., Ltd. (Jiangsu, China). Myofibroblast specific knockout and overexpression mice on *Apoe*^*−/−*^ C57BL/6 background were purchased from GemPharmatech Co., Ltd. (Jiangsu, China). A two-step, 5/6 nephrectomy was applied to establish a CKD mice model. Mice were acclimatized and fed for one week after surgery to observe their condition, and those in better condition were introduced to the next part of the study. The CKD mice model establishing and compound treatment strategy is in Supplementary Methods, the biochemical parameters for the surgical group and sham group are provided in the Fig. S19. Seldom did any of the mice die after surviving 1 week of acclimatization feeding. Detailed construction strategies for gene-edited animals with validation of gene identification were revealed in the Figs. S8-9. Experimental mice were housed in a special pathogen-free, temperature- and light-controlled animal facility under the standard light–dark cycle (12 h/12 h) and allowed ad libitum access to standard rodent chow and water. Mice were anesthetized by 5% isoflurane and euthanized by exsanguination and thoracotomy for sample collection finally. For pharmacological interventions, semaglutide (purchased from Master of Bioactive Molecules) was delivered via subcutaneous injection at a dose of 60 μg/kg/day for 4 weeks. MCC950 (10 mg/kg) was administered via tail vein injection once weekly where indicated. Detailed treatment protocols are provided in the Supplementary Methods.

### Echocardiography

Transthoracic echocardiography was performed as previously described [65].

### Anti-diabetic screening

An anti-diabetic compound library (Selleck, L2900) was screened in VICs under high-phosphate conditions. Cells were treated with compounds (10 nM), and SIRT1/NLRP3 expression was assessed using high-content imaging. Detailed screening procedures are described in the Supplementary Methods.

### Cohorts design for in UKB

Analyses included 275,599 unrelated participants with genetically confirmed White British ancestry from UKB cohort (Fig. S7a). Participants with congenital valvular heart disease (ICD-9 codes 746–747 or ICD-10 codes Q20-Q23) were excluded [66]. Disease definition was determined by in-patient main diagnose, primary death records, and main operation record, in form of International Classification of Diseases (ICD) Ninth Revision and Tenth Revision (field id: 41,202, 40,001) and Classification of Surgical Operations and Procedures (OPCS) Fourth Revision (field id: 41,200), respectively. Full definitions are listed in Table S1. Remaining UKB participants served as controls.

### Statistical analysis

GraphPad Prism 8 (GraphPad Software, Inc., CA, USA) was used to analyze the data. All values were expressed as mean ± standard deviation (SD) for normally distributed data and median (quartile) for else. The normality of distribution of all continuous variables was confirmed by the Shapiro–Wilk test (*n* < 5) or Kolmogorov–Smirnov test (n ≥ 5) and F test was used to compare variances. Normally distributed data with equal variances were analyzed using unpaired two-tailed Student’s t-test (two groups) or one-way ANOVA (≥ 3 groups). Nonparametric data were analyzed by Mann–Whitney U test (two groups) or Kruskal–Wallis test followed by Dunn’s post hoc test (≥ 3 groups). *P* < 0.05 was considered to be statistically significant.

## Supplementary Information

Supplementary Material 1.

## Data Availability

Two single-cell RNA sequencing (scRNA-seq) raw datasets used in this study are available in the NCBI BioProject database under accession number PRJNA562645 (https://ngdc.cncb.ac.cn/bioproject/browse/insdc/PRJNA562645). The remaining scRNA-seq data are available in the National Genomics Data Center database under accession number PRJCA061776 (https://ngdc.cncb.ac.cn/gsub/submit/bioproject/PRJCA061776). Data generated and analyzed in this study are available from the corresponding author upon request, except for UK Biobank data, which are available only through direct application. High-throughput sequencing and analysis protocols are described in the Supplementary Methods.

## Abbreviations

ALP: Alkaline phosphatase
AS: Aortic stenosis
ATP: Adenosine triphosphate
CAVD: Calcific aortic valve disease
CKD: Chronic kidney disease
CRP: C-reactive protein
DEG: Differentially expressed gene
ECAR: Extracellular acidification rate
ELISA: Enzyme-linked immunosorbent assay
eQTL: Expression quantitative trait loci
ESRD: End-stage renal disease
GSEA: Gene set enrichment analysis
GSVA: Gene set variation analysis
HFpEF: Heart failure with preserved ejection fraction
IL-1β: Interleukin-1 beta
IL-18: Interleukin-18
MR: Mendelian randomization
NAD⁺: Nicotinamide adenine dinucleotide
NF-κB: Nuclear factor kappa-B
NLRP3: NLR family pyrin domain-containing 3
OCR: Oxygen consumption rate
OPLS-DA: Orthogonal partial least squares discriminant analysis
OR: Odds ratio
p16: Cyclin-dependent kinase inhibitor 2A
RUNX2: Runt-related transcription factor 2
scRNA-seq: Single-cell RNA sequencing
SD: Standard deviation
SIRT1: Sirtuin 1
UKB: UK Biobank
VIC: Valve interstitial cell
β-Gal: β-Galactosidase
