# Supplementary material for: Sirtuin 1 deficiency mediates chronic kidney disease-induced inflammaging cardiovascular calcification
Source: Mol Biomed. 2026 Jul 7;7:105. doi: 10.1186/s43556-026-00488-3 (PMC13338083; doi:10.1186/s43556-026-00488-3)
Supplement: Supplementary file 1 — Supplementary Material 1. [file 43556_2026_488_MOESM1_ESM.docx]

**Sirtuin 1 Deficiency Mediates Chronic Kidney Disease-Induced Inflammaging Cardiovascular Calcification**

^#^Li Xu, M.D.^1^, ^#^Yidan Zheng, M.D.^1^, ^#^Ming Liu, M.D.^1^, ^#^Bowen Deng^2^, Xingyu Qian, M.D.^1^, Chen Jiang, M.D.^1^, Yuqi Liu, M.D.^1^, Pengning Fan, M.D.^1^, Zhenqi Rao, M.D.^1^, Ming Chen, M.D.^1^, Zhe Chen, M.D.^1^, *Zhejun Cai, M.D.^4^, *Nianguo Dong, M.D.^1^, *Da Zhu, M.D.^3^, *Fei Li, M.D.^1, 3^

^1^Department of Cardiovascular Surgery, Union Hospital, Tongji Medical College, Huazhong University of Science and Technology, 1277 Jiefang Ave., Wuhan, 430022, China

^2^Department of Nephrology, Union Hospital, Tongji Medical College, Huazhong University of Science and Technology, 1277 Jiefang Ave., Wuhan, 430022, China

^3^Department of Cardiac Surgery, Yunnan Fuwai Cardiovascular Hospital, Kunming Medical University, 528 Shahebei Road, 65000 Kunming, China

^4^Department of Cardiology, the Second Affiliated Hospital, Zhejiang University School of Medicine, 88 Jiefang Road, Hangzhou 310009, Zhejiang, China

^#^These authors contributed equally to this work.

Correspondence authors: Fei Li, Da Zhu, Nianguo Dong, Zhejun Cai

*Correspondence to:

Fei Li, ^1^Department of cardiovascular surgery, Union Hospital, Tongji Medical College, Huazhong University of Science and Technology, 1277 Jiefang Ave, Wuhan 430022, China. ^2^Department of Cardiac Surgery, Yunnan Fuwai Cardiovascular Hospital, Kunming Medical University, 528 Shahebei Rd, 65000 Kunming, China. Email: lifei_union@hust.edu.cn

Da Zhu, Structural heart center, Fuwai Yunnan Cardiovascular Hospital, Kunming

Medical University, 528 Shahebei Rd, 65000 Kunming, China. Email: zhuda8687@126.com

Nianguo Dong, Department of cardiovascular surgery, Union Hospital, Tongji Medical College, Huazhong University of Science and Technology, 1277 Jiefang Ave, Wuhan 430022, China. Email: 1986xh0694@hust.edu.cn

Zhejun Cai, Department of Cardiology, the Second Affiliated Hospital, Zhejiang University School of Medicine, 88 Jiefang Road, Hangzhou 310009, Zhejiang, China. Email: caizhejun@zju.edu.cn

**SUPPLEMENTAL MATERIAL**

**SUPPLEMENTAL FIGURES**


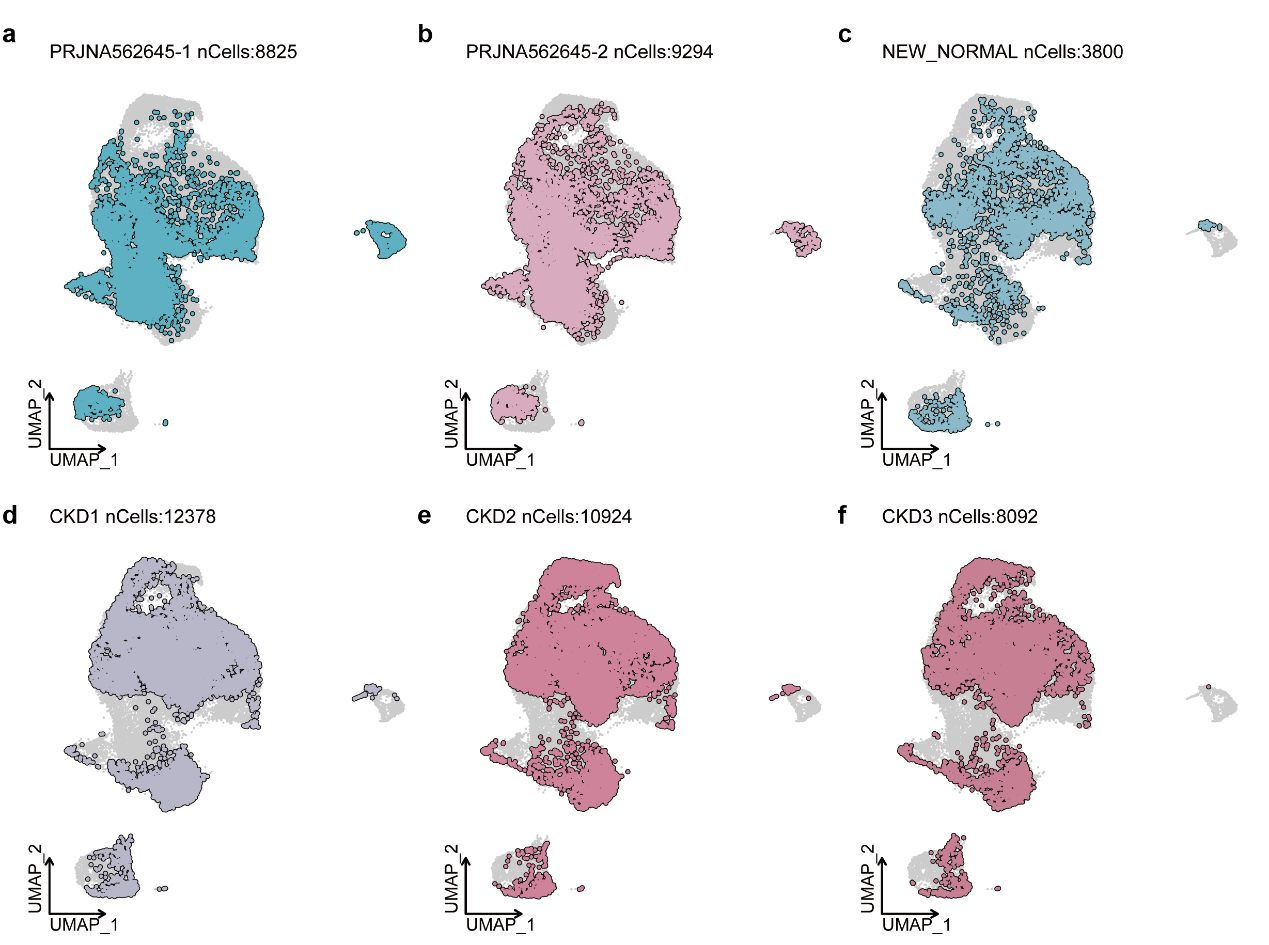


**Fig. S1. Sample idents distribution of scRNA umap dimension reduction plot.**

(**a**) UMAP scRNA reduction plots of NORMAL1 ident. (**b**) UMAP scRNA reduction plots of NORMAL2 ident. (**c**) UMAP scRNA reduction plots of NORMAL3 ident. (**d**) UMAP scRNA reduction plots of CKD1 ident. (**e**) UMAP scRNA reduction plots of CKD2 ident. (**f**) UMAP scRNA reduction plots of CKD3 ident.

**
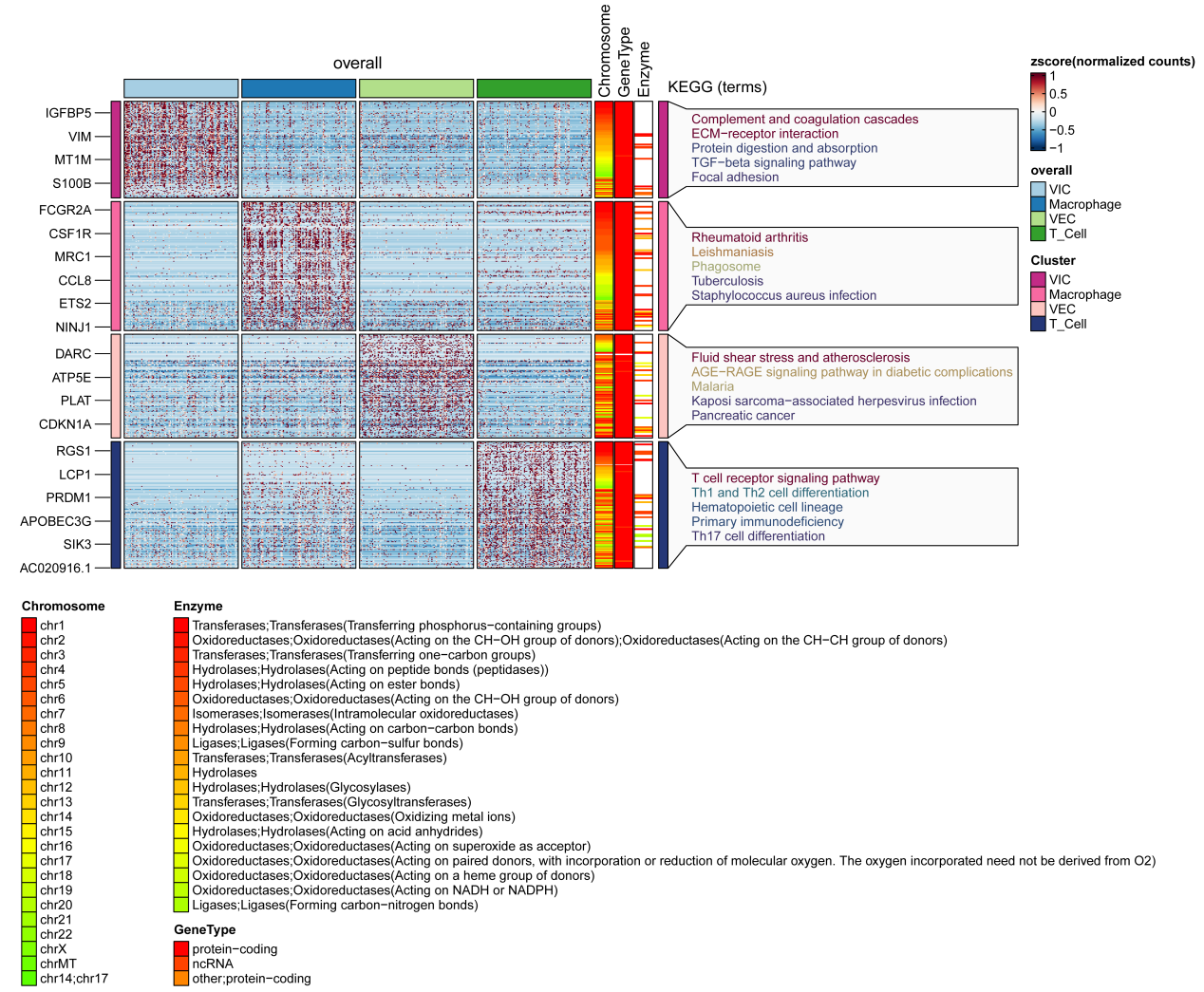
Fig. S2. Heatmap of major cell subpopulations of aortic valves enriched with KEGG pathway.**

**
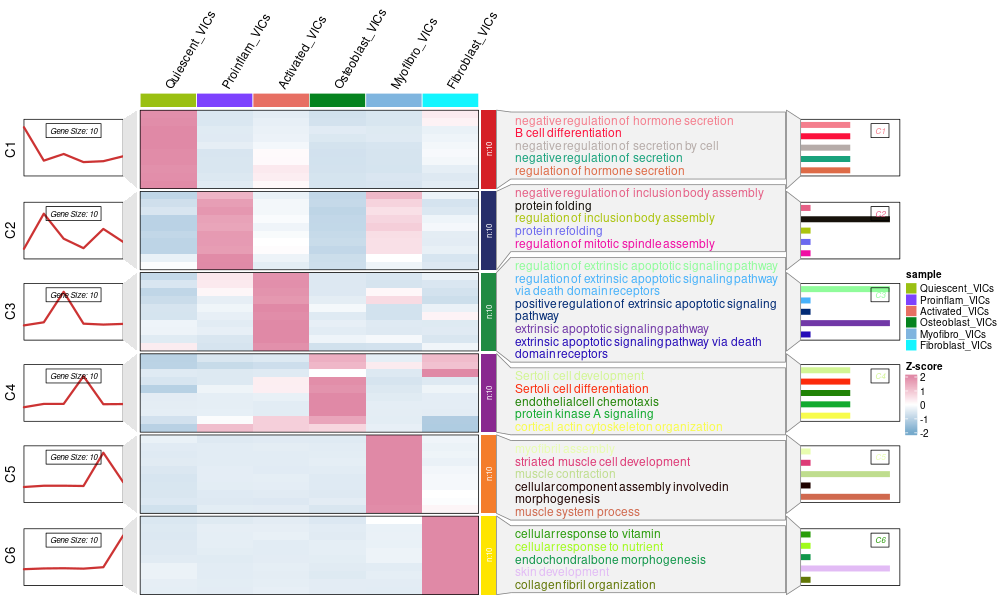
Fig. S3. Heatmap of major cell subpopulations of valvular interstitial cells enriched with KEGG pathway.**


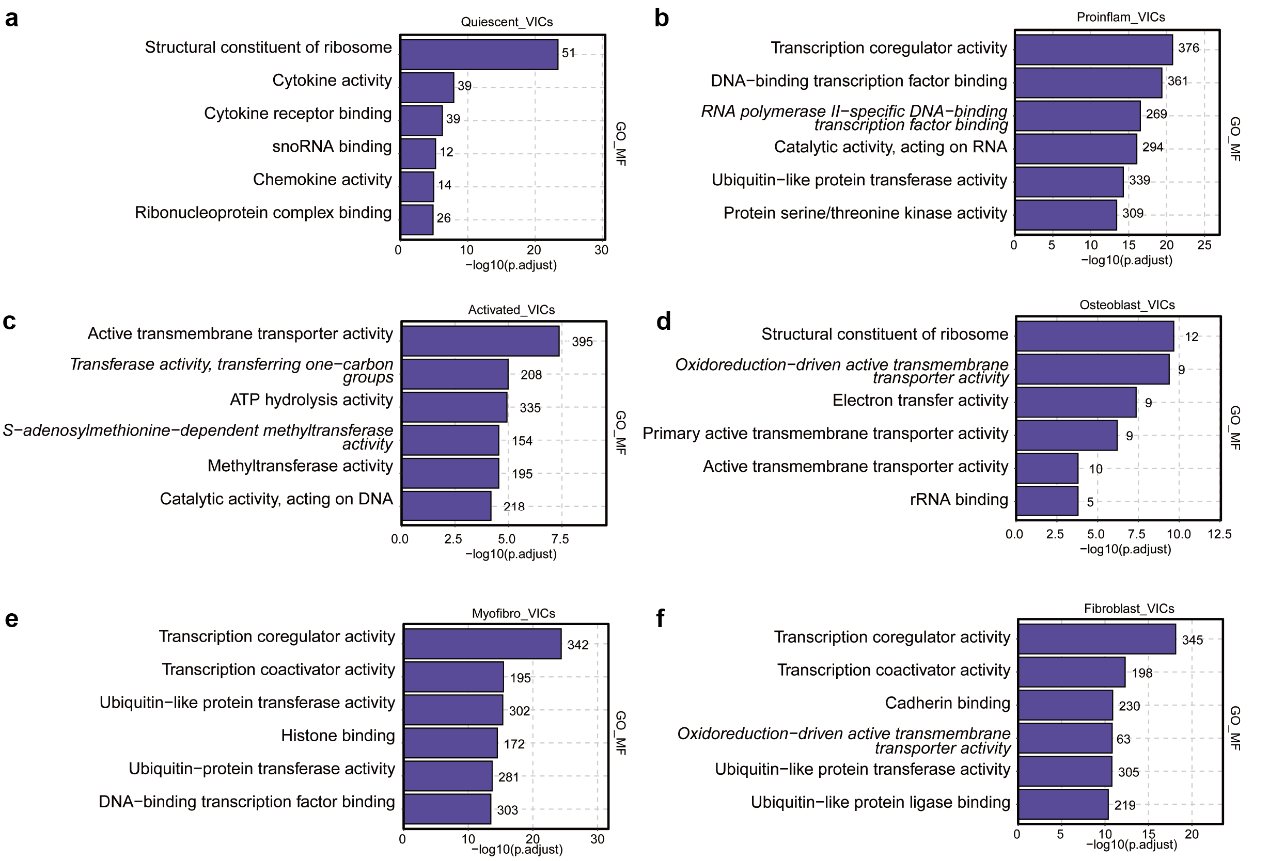


**Fig. S4. GO enrichment map of major subpopulations of valvular interstitial cells.**


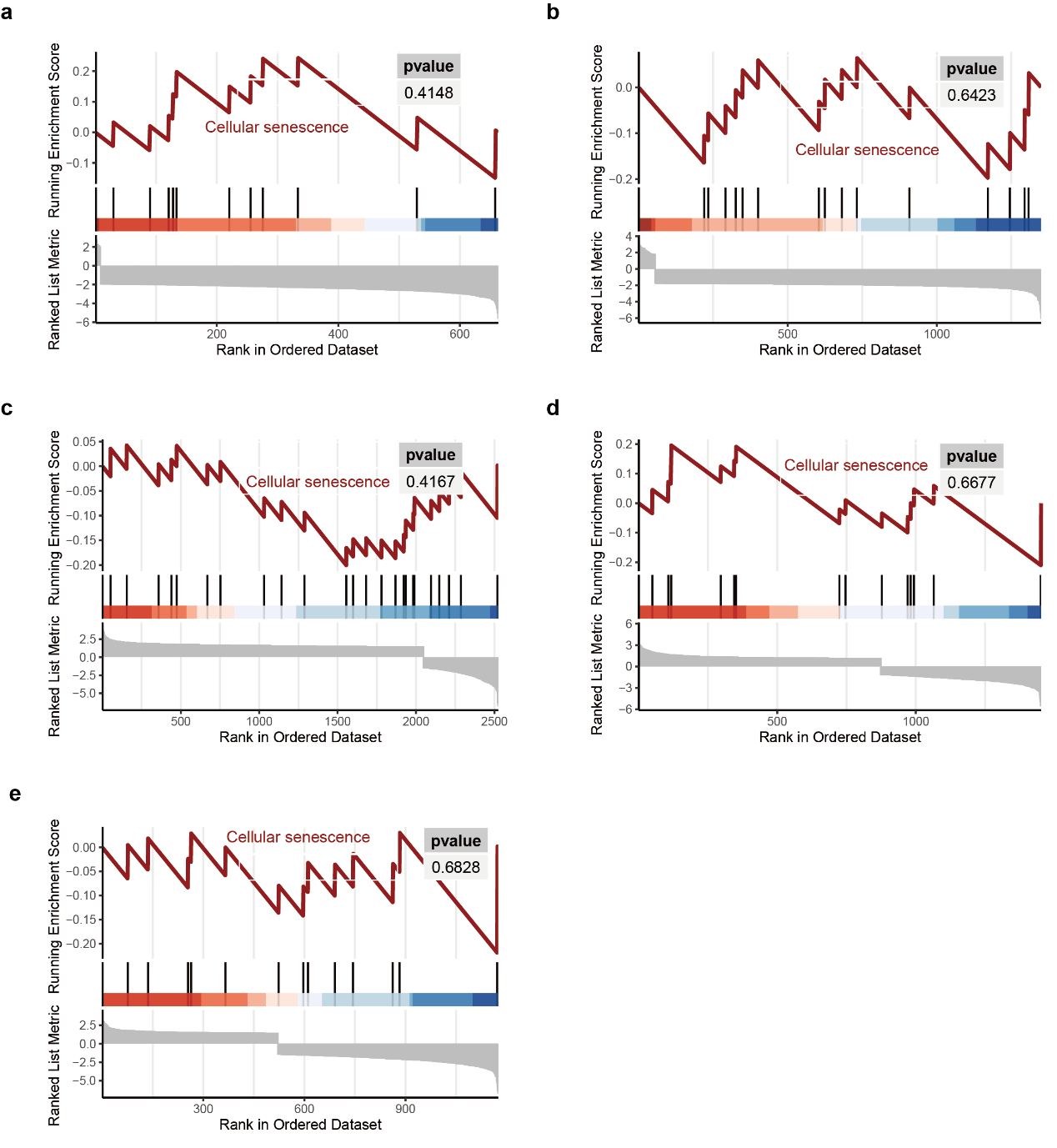


**Fig. S5. GSEA analysis of cellular** **senescence pathways in subpopulations of valvular interstitial cells.**

(**a**) GSEA enrichment of proinflammatory-VICs. (**b**) GSEA enrichment of osteoblast-VICs. (**c**) GSEA enrichment of fibroblast-VICs. (**d**) GSEA enrichment of quiescent-VICs. (**e**) GSEA enrichment of activated-VICs.


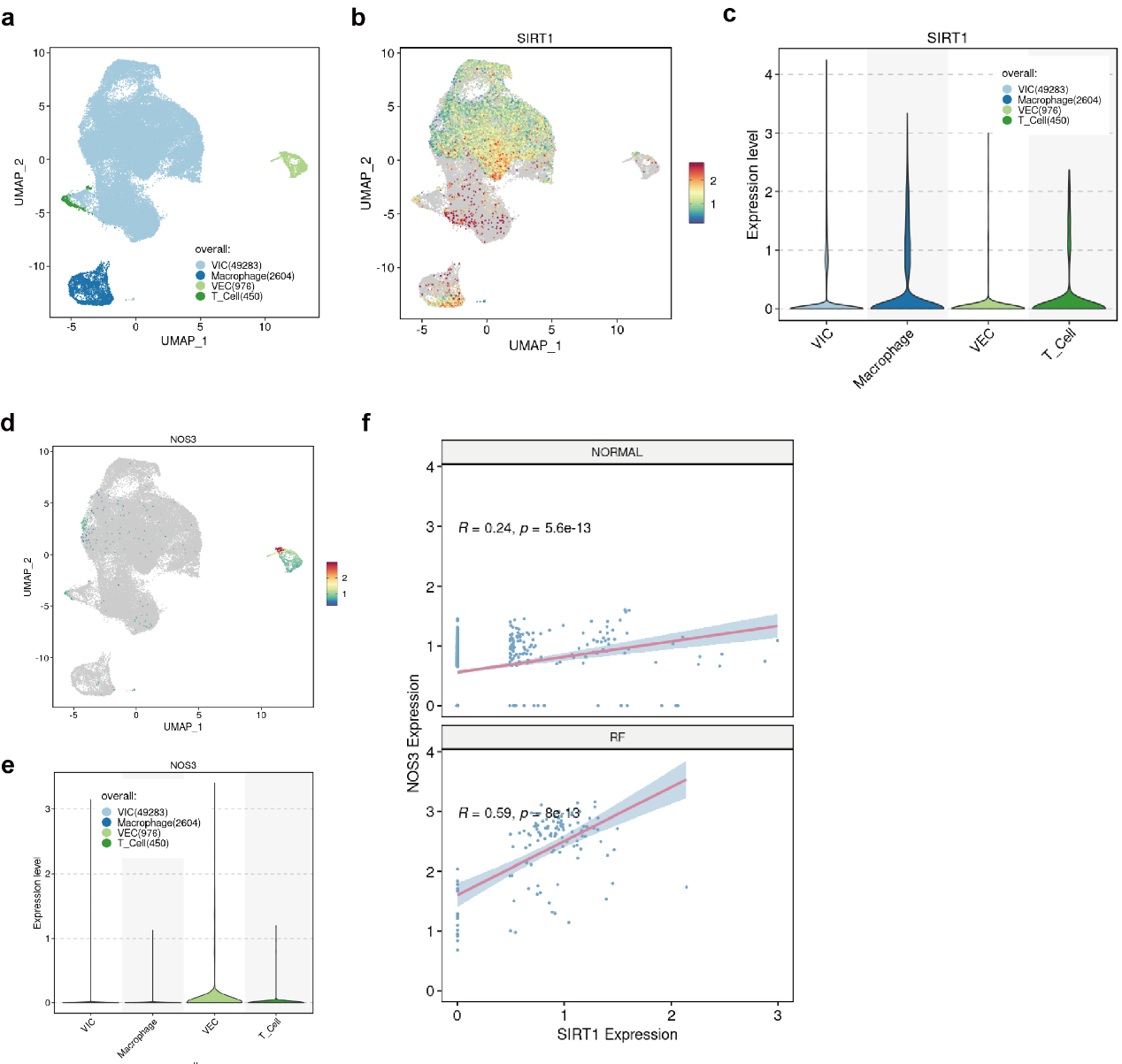


**Fig. S6. scRNA-seq shows SIRT1 mainly in VICs and NOS3 enriched in VECs of human aortic valves.**

**(a)** UMAP of human aortic valve scRNA-seq showing major cell types: VICs, macrophages, VECs, and T cells; **(b)** Feature plot of SIRT1 expression across clusters; **(c)** Violin plot showing SIRT1 is mainly expressed in VICs; **(d)** Feature plot of NOS3 expression across clusters; **(e)** Violin plot showing NOS3 is enriched in VECs; **(f)** Correlation of SIRT1 and NOS3 in endothelial cells from normal valves and calcified aortic valves from CKD patients.


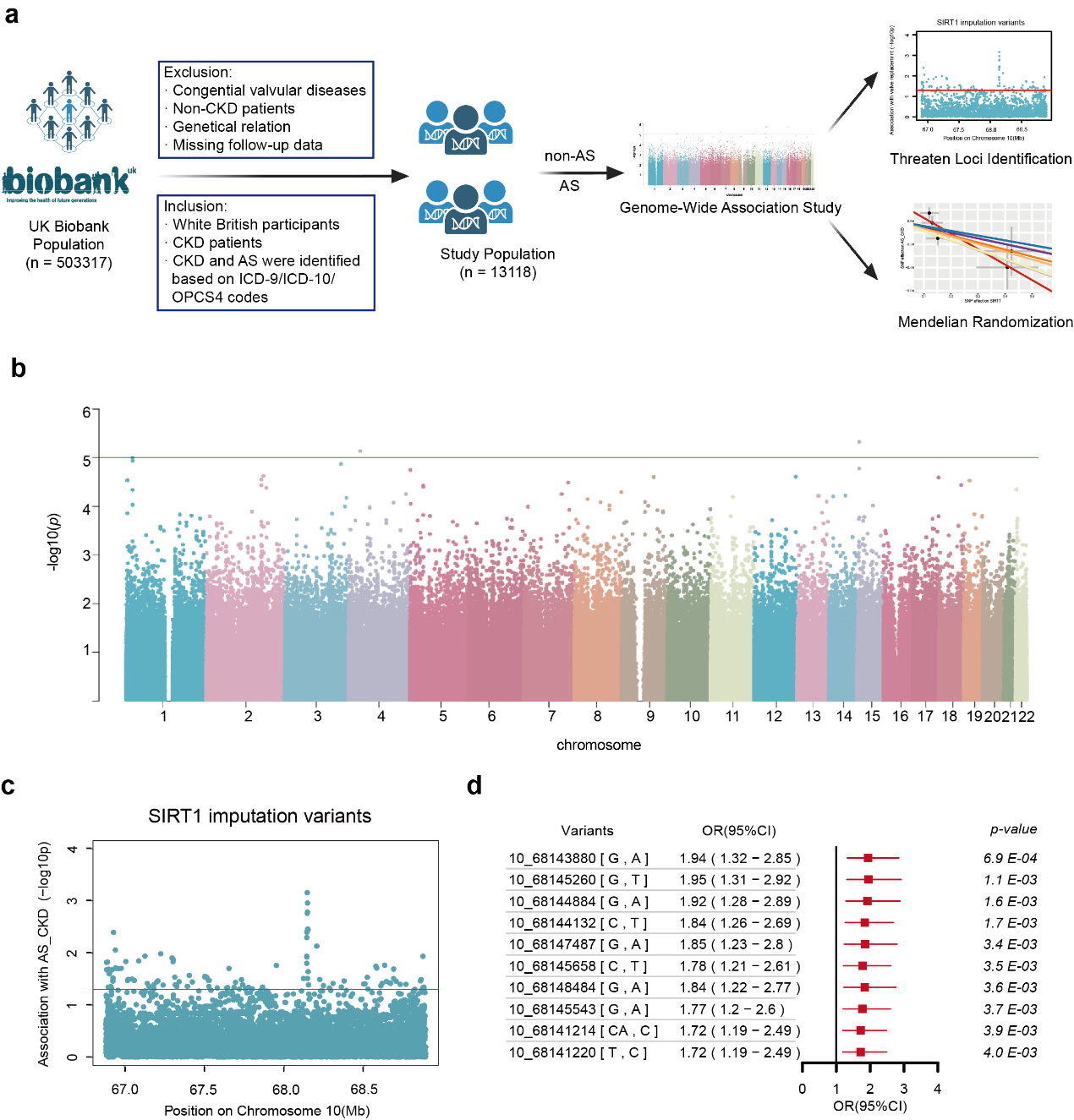


**Fig. S7. Genome-wide analysis revealed association of SIRT1 variants and expression levels with aortic stenosis occurrence in CKD patients.**

(**a**) Schematic diagram of genome analysis and Mendelian randomization based on the UKB population. (**b**) Manhattan plot of genomic variants in patients with AS occurring in the CKD population. (**c**) Manhattan plot of SIRT1 gene region variants in patients with AS occurring in the CKD population. (**d**) Forest plot of association with aortic stenosis of variants in SIRT1 gene.


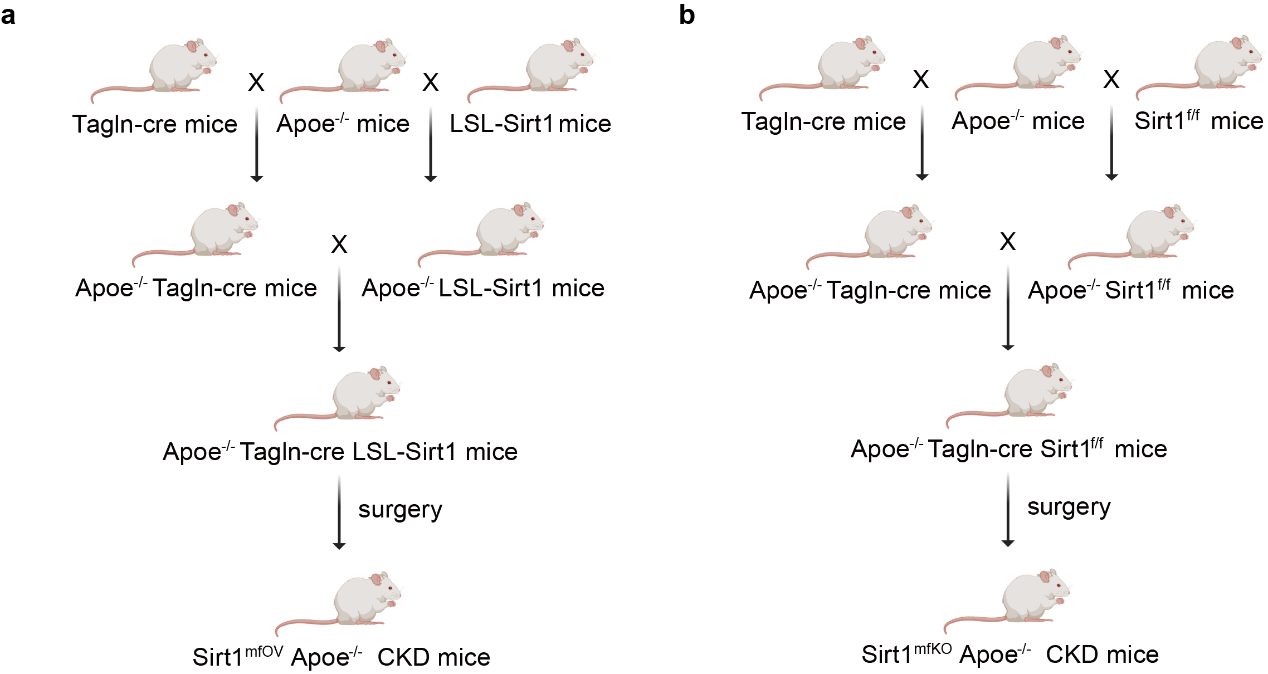


**Fig. S8. Detailed constructing strategy of mice.**

(**a-b**) Constructing strategies of *Sirt1^mfOV^ Apoe^-/-^* mice and *Sirt1^mfKO^ Apoe^-/-^* mice.

**
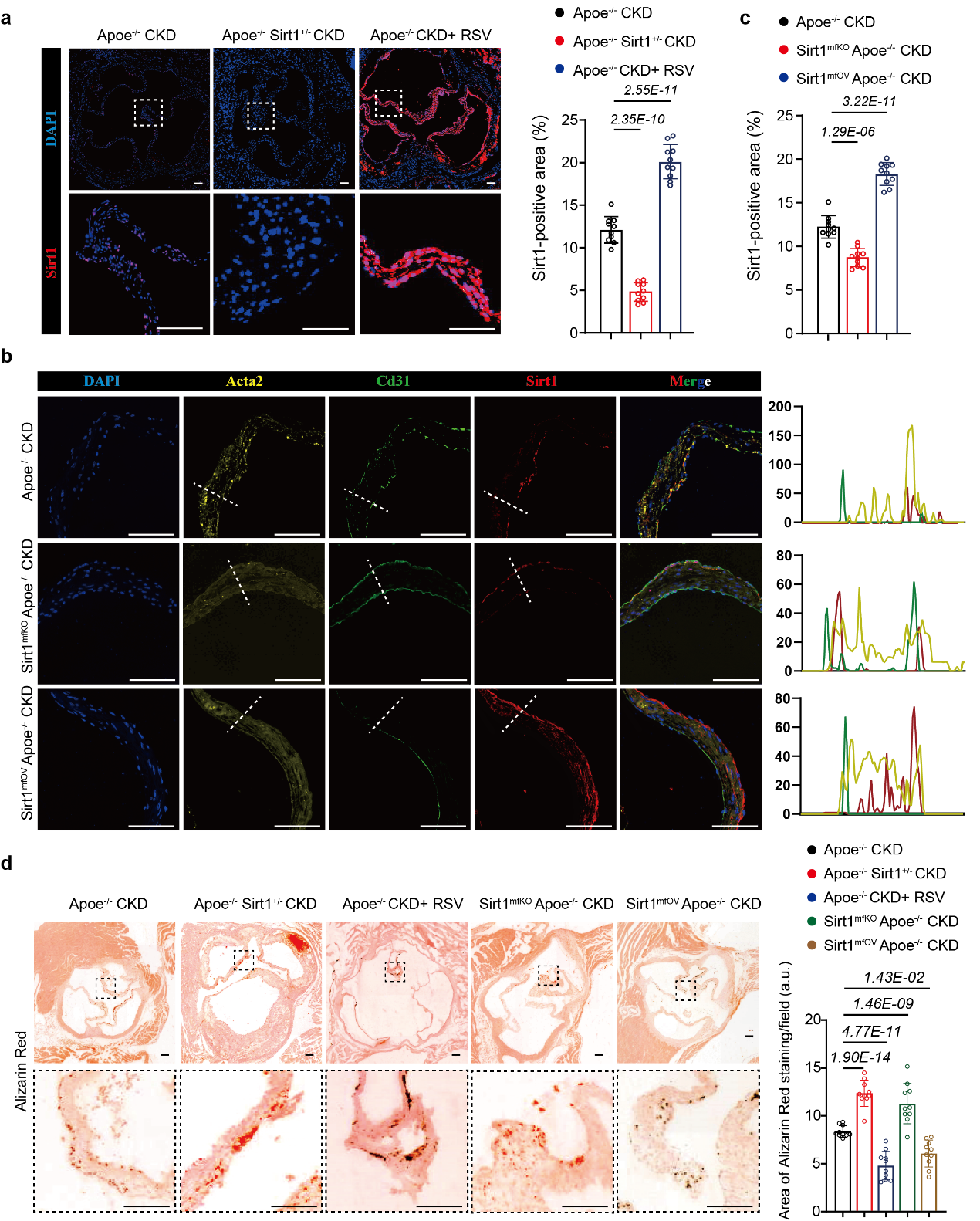
**

**Fig. S9. Gene editing validation of mice.**

**(a)** Representative immunofluorescence images and quantification of Sirt1 expression in aortic valves. Compared with *Apoe^⁻/⁻^* CKD mice, Sirt1 expression (red) was significantly decreased in *Apoe^⁻/⁻^* *Sirt1*^⁺/⁻^ CKD mice and markedly increased following resveratrol (RSV) treatment. Nuclei were counterstained with DAPI (blue). Scale bar = 100 μm. (**b-c)** Representative multichannel immunofluorescence images and corresponding quantitative analysis of Sirt1 expression in genetically modified models. Staining includes DAPI (blue, nuclei), Acta2 (yellow, smooth muscle marker), Cd31 (green, endothelial marker), and Sirt1 (red), along with merged images to illustrate spatial colocalization within the valve structure. Compared with *Apoe^⁻/⁻^* CKD mice, Sirt1 expression was markedly reduced in *Sirt1^mfKO^* *Apoe^⁻/⁻^* CKD mice and significantly increased in *Sirt1^mfOV^* *Apoe^⁻/⁻^* CKD mice. Line-scan intensity profiles further demonstrate the distribution and relative signal intensity of each channel across the valve leaflet. Scale bar = 100 μm. **(d)** Representative Alizarin Red S staining and quantitative analysis of aortic valve calcification. Calcium deposition was prominently detected in *Apoe^⁻/⁻^* CKD mice and further increased in Sirt1-deficient conditions, whereas RSV treatment or Sirt1 overexpression markedly reduced calcification. In contrast, *Sirt1^mfKO^* mice exhibited persistent and pronounced calcification. Positive staining appeared as localized deposits within valve tissue rather than diffuse background signals, supporting specific detection of calcium accumulation. Scale bar = 100 μm.


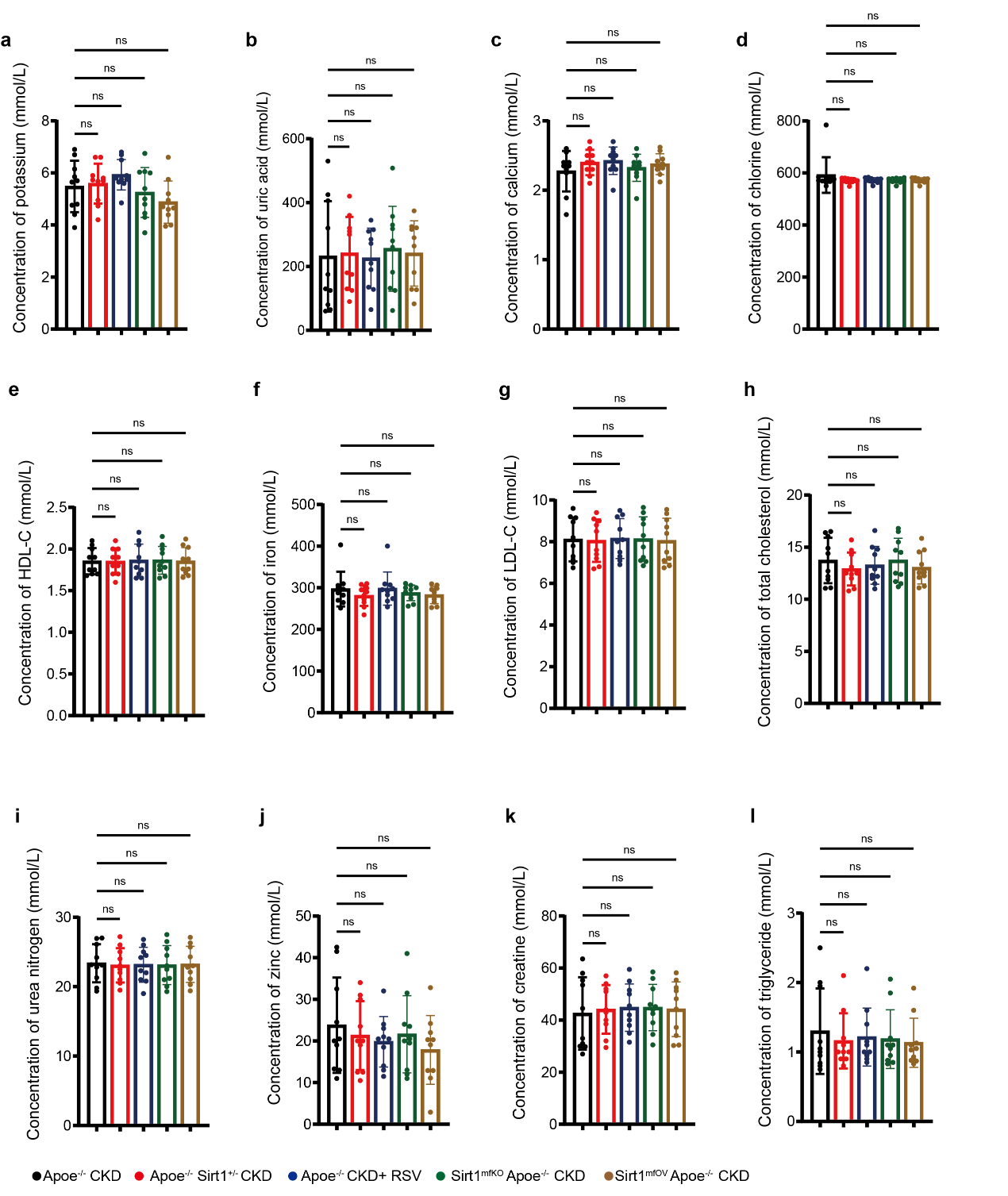


**Fig. S10. Biochemical parameters in the mouse models of chronic kidney disease.**

(**a**) Plasma concentration of potassium in the mice models. (**b**) Plasma concentration of uric acid in the mice models. (**c**) Plasma concentration of calcium in the mice models. (**d**) Plasma concentration of chlorine in the mice models. (**e**) Plasma concentration of HDL-C in the mice models. (**f**) Plasma concentration of iron in the mice models. (**g**) Plasma concentration of LDL-C in the mice models. (**h**) Plasma concentration of total cholesterol in the mice models. (**i**) Plasma concentration of urea nitrogen in the mice models. (**j**) Plasma concentration of zinc in the mice models. (**k**) Plasma concentration of creatinine in the mice models. (**l**) Plasma concentration of triglyceride in the mice models.


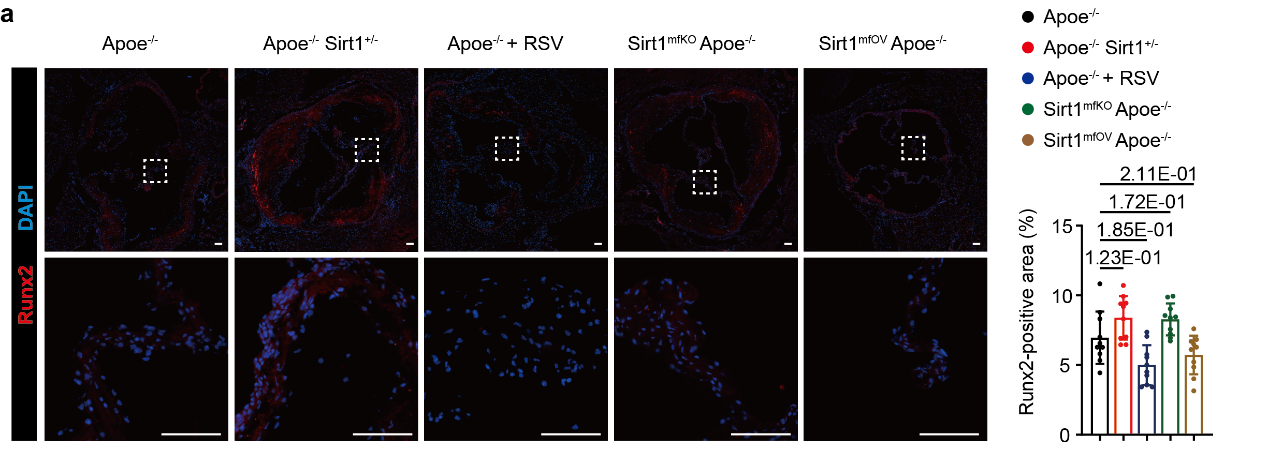


**Fig. S11. Runx2 expression in aortic valves under non-CKD (sham) conditions.**

Representative immunofluorescence staining and quantification of Runx2 expression in aortic valve sections from *Apoe^⁻/⁻^, Apoe^⁻/⁻^ Sirt1^⁺/⁻^, Apoe⁻/⁻* + RSV, *Sirt1^mfKO^* *Apoe*^⁻/⁻^, and *Sirt1^mfOV^* *Apoe⁻/⁻* mice under sham conditions. Runx2 (red) and nuclei (DAPI, blue) are shown. Dashed boxes indicate regions selected for higher magnification. Quantification of Runx2-positive area (%) is presented on the right, with each dot representing an individual mouse and data shown as mean ± SEM. Scale bar = 100 μm.


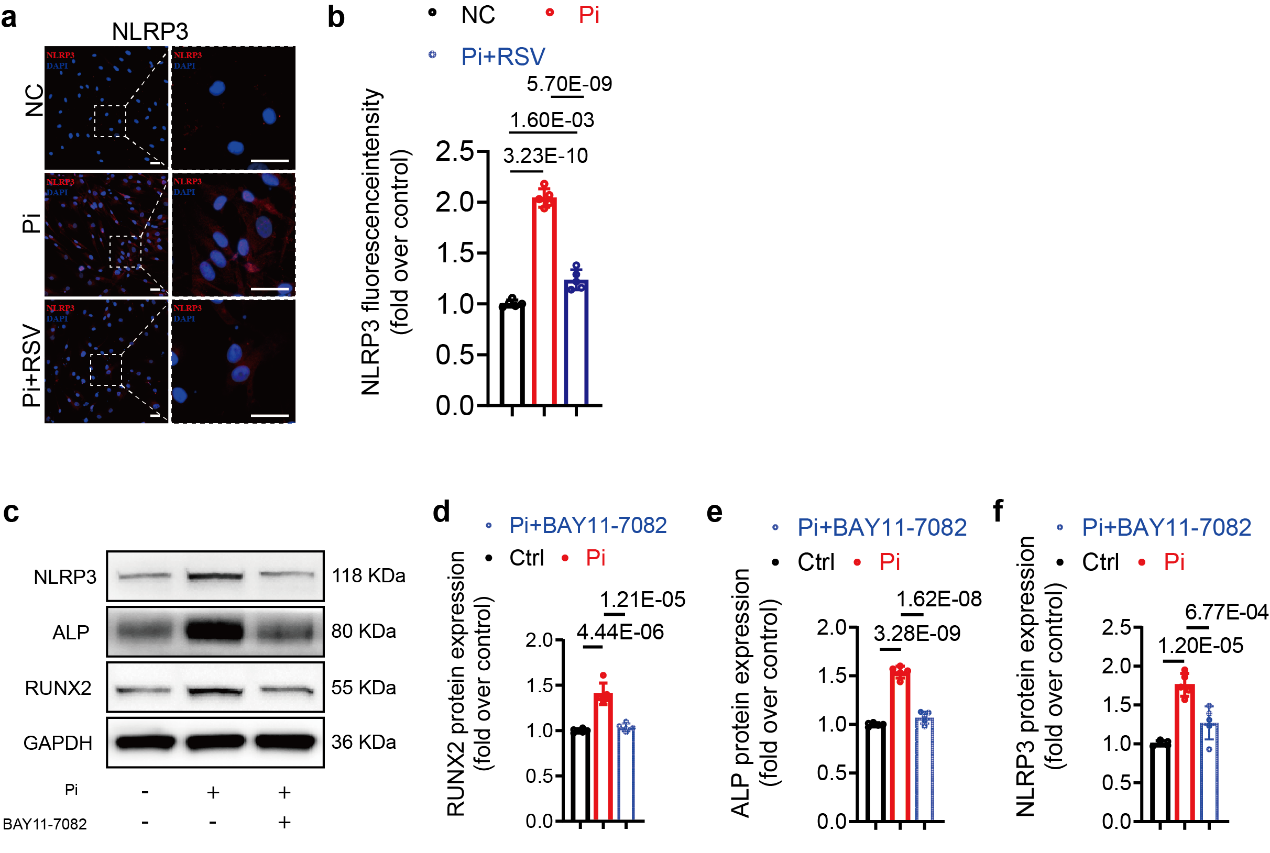


**Fig. S12. SIRT1 activation and NF-κB inhibition attenuated NLRP3 activation *in vitro*.**

(**a-b)** resveratrol treated VICs had significantly lower expression of NLRP3 (red), DAPI (blue) was used for nuclear counterstaining. Scale bar = 25 μm. (**c-f**) Representative western blot band and densitometric quantification showed that NF-κB pathway/IκBα phosphorylation inhibitor BAY11-7082 significantly decreased NLRP3, ALP and RUNX2 expression in VICs treated with hyperphosphate culture medium for 72h.


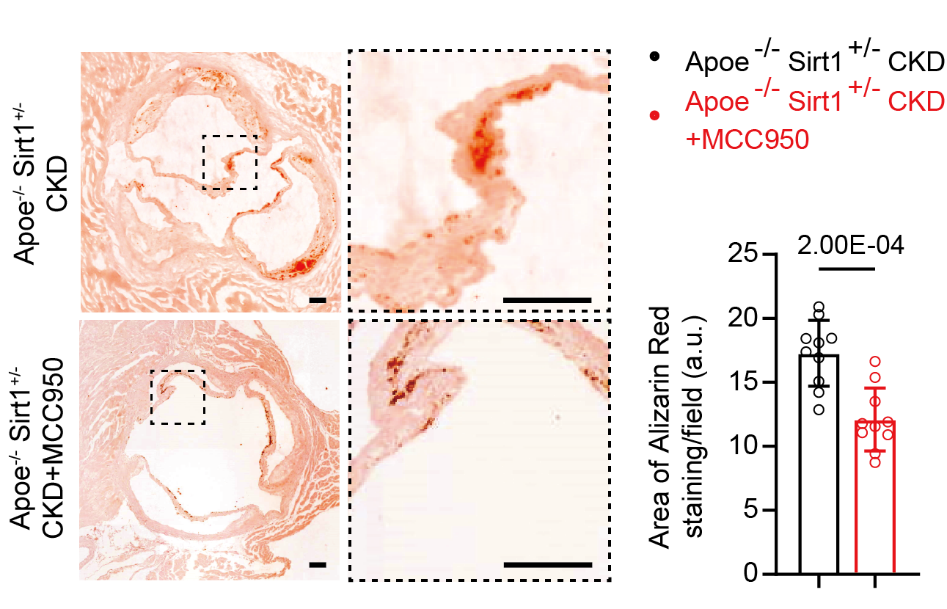


**Fig. S13. Effects of MCC950 treatment on aortic valve calcification in CKD mice.**

Representative Alizarin Red S staining and quantification of calcium deposition in aortic valves from *Apoe^⁻/⁻^* *Sirt1^⁺/⁻^* CKD mice with or without MCC950 treatment. Dashed boxes indicate regions shown at higher magnification. Quantification of Alizarin Red-positive area is shown on the right. Scale bar = 100 μm.


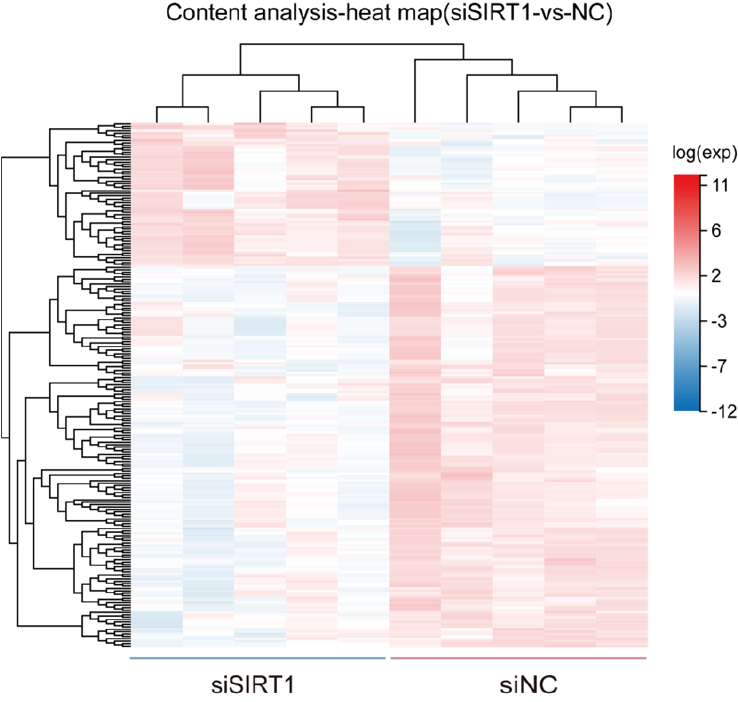


**Fig. S14. Heatmap of the metabolome of valve interstitial cells with knockdown of the SIRT1 compared with control.**


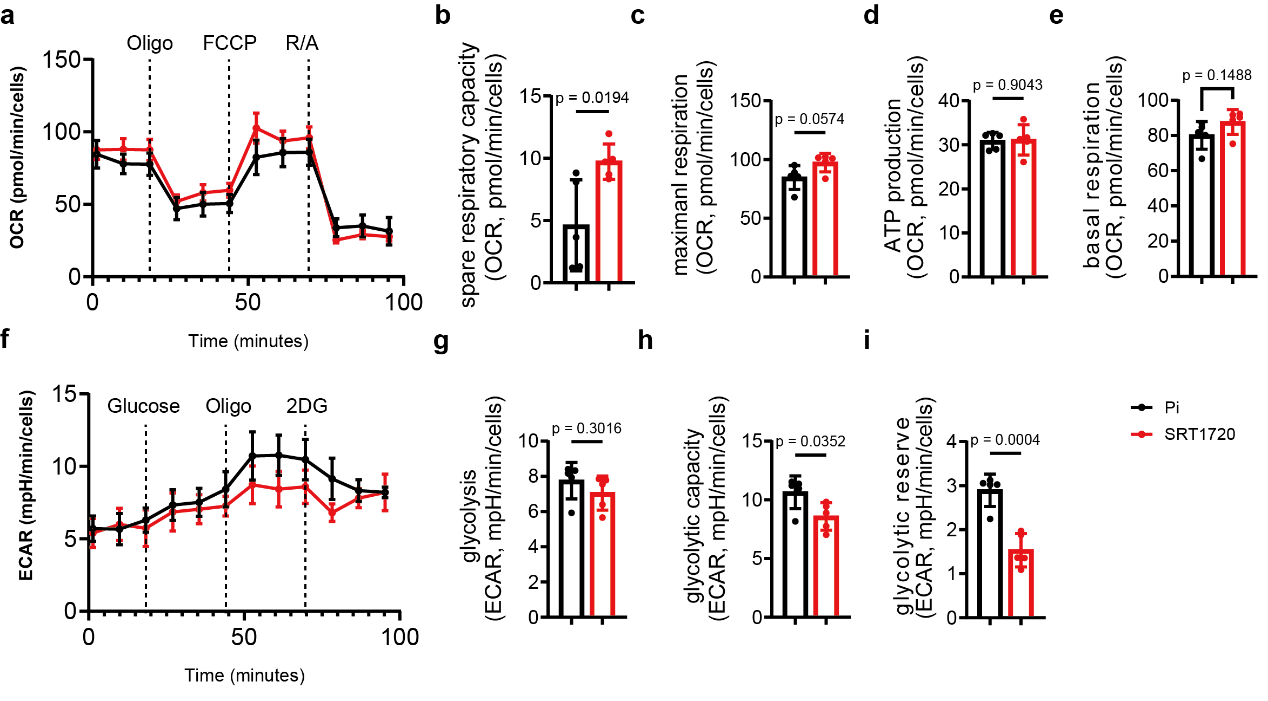


**Fig. S15. Real-time changes in the OCR and ECAR of VICs, pretreated with SRT1720 or Pi, measured using Seahorse.**

Oligo, oligomycin; FCCP, carbonyl cyanide4-(trifluoromethoxy) phenylhydrazone; R/A, rotenone plus antimycin A; 2-DG, 2-deoxy-d-glucose. basal respiration, spare respiratory capacity, maximal respiration, and ATP production were determined based on OCR readings. Glycolytic reserve, glycolysis, and glycolytic capacity were extracted from ECAR.


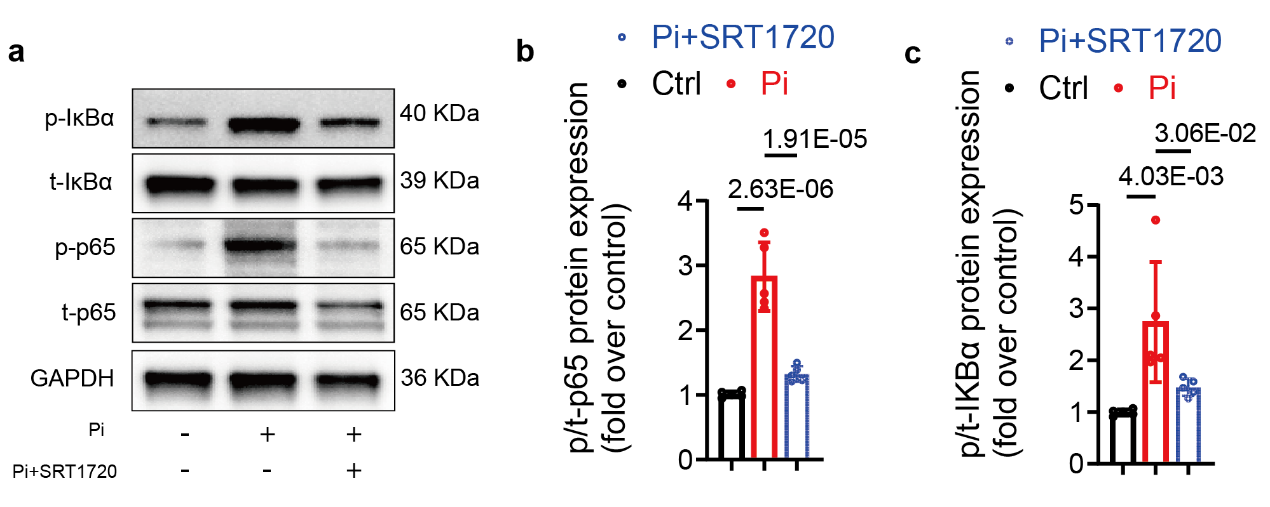


**Fig. S16. SIRT1 activation suppressed NF-κB phosphorylation *in vitro*.**

(**a-c**) Representative western blot band and densitometric quantification showed that SIRT1 agonist SRT1720 significantly decreased p/t-p65 and p/t-IKBα levels in VICs treated with hyperphosphate culture medium for 72h.


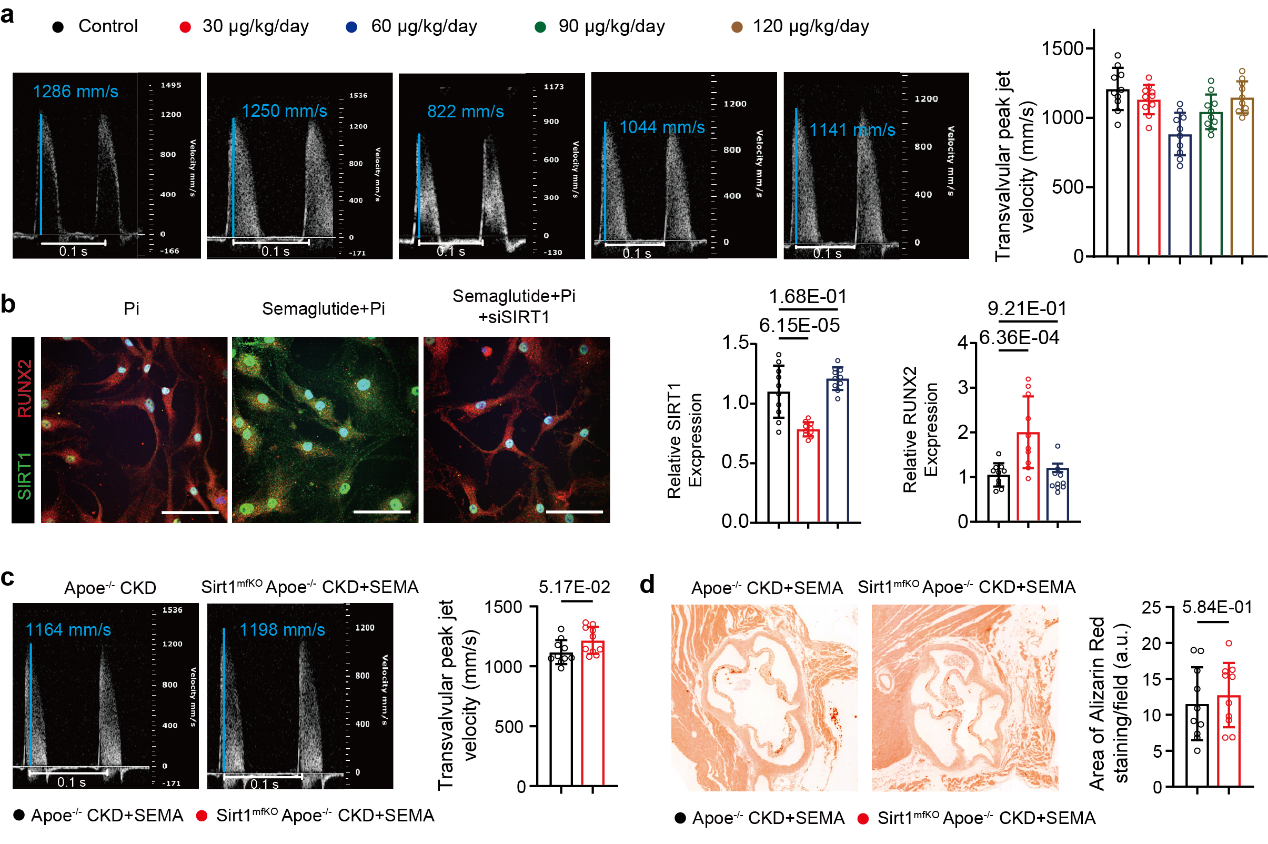


**Fig. S17. Dose-range exploration and validation of the role of SIRT1 in semaglutide-mediated protection against CKD-associated aortic valve calcification.**(**a**) Representative Doppler echocardiographic images and quantification of transvalvular peak jet velocity in the indicated groups treated with different doses of semaglutide (30, 60, 90, and 120 μg/kg/day) or control. These experiments were performed to explore the feasible dose range of semaglutide *in vivo*. (**b**) Representative immunofluorescence staining and quantification of SIRT1 and RUNX2 expression in valvular interstitial cells cultured under phosphate (Pi) conditions, with semaglutide treatment in the presence or absence of siSIRT1. Nuclei were counterstained with DAPI. Semaglutide increased SIRT1 expression and reduced osteogenic marker expression, whereas SIRT1 silencing attenuated these effects. Scale bar = 50 μm. (**c**) Representative Doppler echocardiographic images and quantification of transvalvular peak jet velocity in *Apoe^⁻/⁻^* CKD+SEMA mice and *Sirt1^mfKO^* *Apoe^⁻/⁻^* CKD+SEMA mice. The improvement in hemodynamic valve function observed with semaglutide was abolished in Sirt1-deficient mice. (**d**) Representative Alizarin Red staining and quantification of calcified area in aortic valves from *Apoe^⁻/⁻^* CKD+SEMA mice and *Sirt1^mfKO^* *Apoe^⁻/⁻^* CKD+SEMA mice. The anticalcific effect of semaglutide was lost after Sirt1 deletion. Data are presented as mean ± SD with individual values shown. Exact P values are indicated in the graphs. Scale bar = 100 μm.


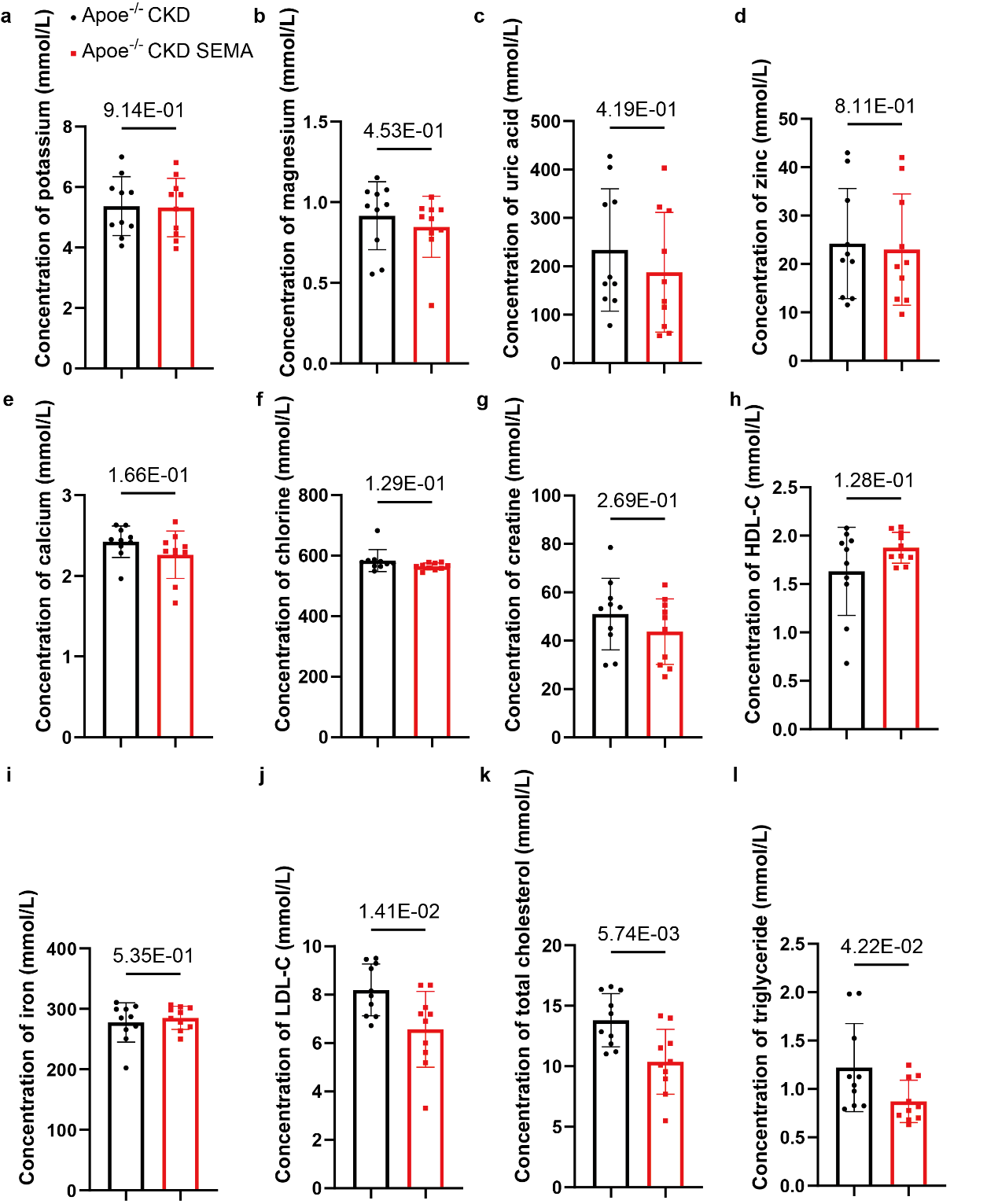


**Fig. S18. Systematic assessment of plasma biochemical parameters in *Apoe^-/-^* CKD mice with or without semaglutide treatment.**(**a**) Plasma concentration of potassium in the mice model. (**b**) Plasma concentration of magnesium in the mice model. (**c**) Plasma concentration of uric acid in the mice model. (**d**) Plasma concentration of zinc in the mice model. (**e**) Plasma concentration of calcium in the mice model. (**f**) Plasma concentration of chlorine in the mice model. (**g**) Plasma concentration of creatinine in the mice model. (**h**) Plasma concentration of HDL-C in the mice model. (**i**) Plasma concentration of iron in the mice model. (**j**) Plasma concentration of LDL-C in the mice model. (**k**) Plasma concentration of total cholesterol in the mice model. (**l**) Plasma concentration of triglyceride in the mice model.


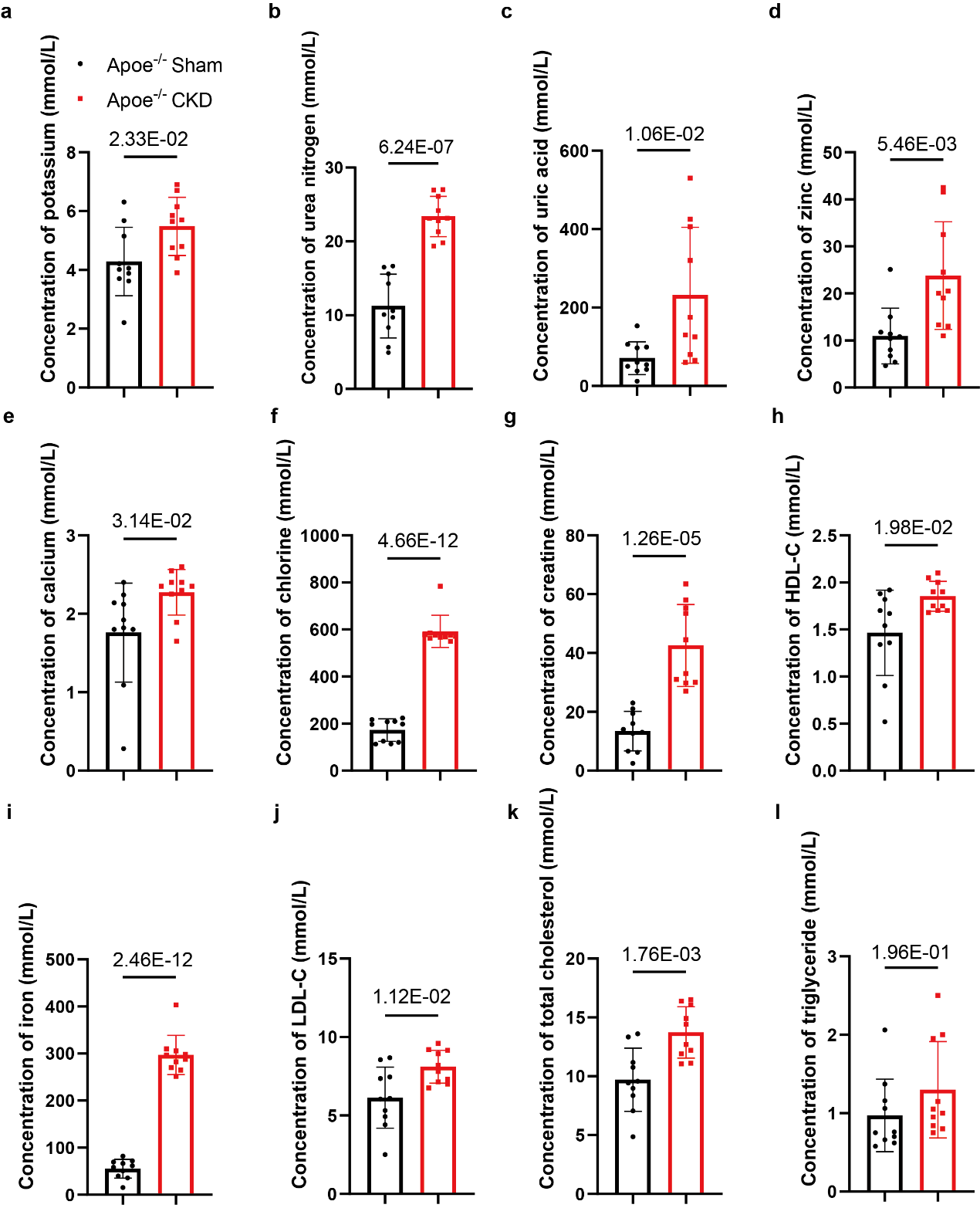


**Fig. S19. Biochemical parameters in the mouse model of chronic kidney disease.**

(**a**) Plasma concentration of potassium in the CKD mice model. (**b**) Plasma concentration of urea nitrogen in the CKD mice model. (**c**) Plasma concentration of uric acid in the CKD mice model. (**d**) Plasma concentration of zinc in the CKD mice model. (**e**) Plasma concentration of calcium in the CKD mice model. (**f**) Plasma concentration of chlorine in the CKD mice model. (**g**) Plasma concentration of creatinine in the CKD mice model. (**h**) Plasma concentration of HDL-C in the CKD mice model. (**i**) Plasma concentration of iron in the CKD mice model. (**j**) Plasma concentration of LDL-C in the CKD mice model. (**k**) Plasma concentration of total cholesterol in the CKD mice model. (**l**) Plasma concentration of triglyceride in the CKD mice model.

**Table S1. Clinical definition in cohort of UK Biobank.**

| Phenotype | Source | Code |
| --- | --- | --- |
| Aortic stenosis | ICD-9 | 424.1 |
|  | ICD-10 | I35.0 |
|  | OPCS4 | K26.1, K26.2, K26.3, K26.4, K31.2, K32.2, K35.2 |
| Congenital heart disease | ICD-9 | 746, 747 |
|  | ICD-10 | Q20, Q21, Q22, Q23 |
| Chronic kidney failure | ICD-9 | 585 |
|  | ICD-10 | N18 |

**Table S2. Clinical characters of aortic valve donors.**

|  | CAVD | Control | p |
| --- | --- | --- | --- |
| n | 20 | 20 |  |
| Age | 58.50 [53.75, 62.25] | 55.00 [52.00, 61.25] | 0.551 |
| Female | 5 (25.0) | 7 (35.0) | 0.73 |
| Male | 15 (75.0) | 13 (65.0) |  |
| BMI | 21.72 [20.44, 23.83] | 22.46 [20.53, 23.68] | 0.85 |
| Systolic_blood_pressure | 135.00 [127.00, 144.00] | 103.50 [94.25, 111.75] | <0.001 |
| Aortic_valve_orifice_flow_rate | 4.00 [3.15, 4.73] | 1.55 [1.40, 1.92] | <0.001 |
| Red_blood_cell | 3.46 [3.05, 3.86] | 4.51 [4.20, 4.81] | <0.001 |
| Uric_acid | 430.35 [339.68, 527.60] | 325.90 [268.00, 439.08] | 0.023 |
| Creatinine | 131.25 [80.98, 521.58] | 85.10 [76.20, 95.60] | 0.027 |
| Urea_nitrogen | 10.44 [8.15, 19.54] | 7.41 [4.74, 8.97] | 0.003 |
| Potassium | 4.44 [3.97, 4.82] | 4.22 [4.02, 4.45] | 0.176 |

**Table S3. Basic information of the samples used for sequencing from CKD-CAVD donor.**

|  | CKD1 | CKD2 | CKD3 |
| --- | --- | --- | --- |
| Age | 61 | 53 | 57 |
| Gender | female | male | male |
| BMI | 21.56 | 24.88 | 22.14 |
| Systolic_blood_pressure | 130 | 141 | 137 |
| Aortic_valve_orifice_flow_rate | 3.77 | 4.53 | 3.80 |
| Red_blood_cell | 3.28 | 3.73 | 3.49 |
| Uric_acid | 623.43 | 593.66 | 498.38 |
| Creatinine | 449.13 | 203.42 | 248.19 |
| Urea_nitrogen | 37.92 | 16.81 | 25.76 |
| Potassium | 4.92 | 4.16 | 4.02 |

**Table S4. Variants applied in Mendelian Randomization analysis.**

Associations With *SIRT1* Expression and With Aortic Stenosis (for Variants in the Mendelian Randomization Instrument for Aortic Stenosis)

| SNP | effect_allele.  exposure | other_allele.  exposure | effect_allele.  outcome | other_allele.  outcome | beta.  exposure | beta.  outcome | se.  outcome | pval.  outcome | se.exposure | pval.exposure |
| --- | --- | --- | --- | --- | --- | --- | --- | --- | --- | --- |
| rs10822957 | T | C | T | C | -0.15130669 | 0.148049247 | 0.0614771 | 0.0160333 | 0.043029765 | 0.000512499 |
| rs10823139 | A | T | A | T | 0.13109675 | -0.013992439 | 0.0597674 | 0.814898 | 0.038986812 | 0.00088303 |
| rs10997815 | T | C | T | C | 0.12026811 | 0.069712612 | 0.0517005 | 0.17754 | 0.034454894 | 0.000562113 |
| rs113700470 | C | A | C | A | -0.4240699 | 0.25896619 | 0.212362 | 0.222671 | 0.125460248 | 0.000830748 |
| rs148377355 | G | T | G | T | -0.408477 | 0.39853448 | 0.188596 | 0.0345861 | 0.114732956 | 0.000437181 |

Associations With *SIRT1* Expression and Aortic Valve Replacement (for Variants in the Mendelian Randomization Instrument for Aortic Valve Replacement)

| SNP | effect_allele.  exposure | other_allele.  exposure | effect_allele.  outcome | other_allele.  outcome | beta.  exposure | beta.  outcome | se.  outcome | pval.  outcome | se.exposure | pval.exposure |
| --- | --- | --- | --- | --- | --- | --- | --- | --- | --- | --- |
| rs10822957 | T | C | T | C | -0.15130669 | 0.117774147 | 0.123612 | 0.340713 | 0.043029765 | 0.000512499 |
| rs10823139 | A | T | A | T | 0.13109675 | -0.100025881 | 0.118565 | 0.398868 | 0.038986812 | 0.00088303 |
| rs10997815 | T | C | T | C | 0.12026811 | -0.078270778 | 0.106343 | 0.461714 | 0.034454894 | 0.000562113 |
| rs113700470 | C | A | C | A | -0.4240699 | -0.053736276 | 0.422274 | 0.898739 | 0.125460248 | 0.000830748 |
| rs148377355 | G | T | G | T | -0.408477 | 0.524675273 | 0.352275 | 0.136384 | 0.114732956 | 0.000437181 |

Associations With *NLRP3* Expression and Aortic Valve Replacement (for Variants in the Mendelian Randomization Instrument for Aortic Valve Replacement)

| SNP | effect_allele.  exposure | other_allele.  exposure | effect_allele.  outcome | other_allele.  outcome | beta.  exposure | beta.  outcome | se.  outcome | pval.  outcome | se.exposure | pval.exposure |
| --- | --- | --- | --- | --- | --- | --- | --- | --- | --- | --- |
| rs111487487 | T | C | T | C | 0.5713834 | 0.485692414 | 0.518364 | 0.348774 | 0.216696069 | 0.008850549 |
| rs111980491 | C | T | C | T | 0.247006 | 0.157755603 | 0.177735 | 0.374751 | 0.090339298 | 0.006663661 |
| rs12407904 | T | C | T | C | -0.6496988 | -0.647034884 | 0.397792 | 0.103829 | 0.192113766 | 0.000825745 |
| rs141780770 | C | T | C | T | 0.8665689 | -0.153524209 | 0.454381 | 0.735456 | 0.253677179 | 0.000732478 |
| rs17719892 | G | A | G | A | 0.5867915 | -0.458759878 | 0.378904 | 0.22599 | 0.197316553 | 0.003204593 |
| rs4654292 | G | A | G | A | -0.1758591 | -0.215954455 | 0.119067 | 0.069721 | 0.06057893 | 0.003999274 |
| rs4971321 | G | A | G | A | 0.24278921 | -0.071202812 | 0.145197 | 0.623861 | 0.085376305 | 0.004796928 |
| rs7367735 | C | T | C | T | -0.46541315 | -2.084928568 | 0.905398 | 0.0212914 | 0.174959695 | 0.008275155 |
| rs74227714 | G | A | G | A | 0.2605472 | 0.092670335 | 0.164938 | 0.5742 | 0.091254459 | 0.004632697 |
| rs76475555 | G | C | G | C | 0.58239764 | 0.123111039 | 0.284567 | 0.665278 | 0.202440449 | 0.004334461 |
| rs78789435 | A | C | A | C | -0.25738022 | -0.320382966 | 0.203805 | 0.11595 | 0.098003339 | 0.009122658 |

**Table S5. Anti-diabtets chemicals list in high-content screening.**

| **Name** | **Formulation** | **Target** | **Pathway** | **M.w.** | **CAS Number** | **Formula** |
| --- | --- | --- | --- | --- | --- | --- |
| SRT1720 HCl | in 10mM DMSO | Sirtuin | Epigenetics | 506.02 | 1001645-58-4 | C25H24ClN7OS |
| MK-8245 | in 10mM DMSO | Dehydrogenase | Metabolism | 467.25 | 1030612-90-8 | C17H16BrFN6O4 |
| CHIR-99021 (CT99021) | in 10mM DMSO | GSK-3 | PI3K/Akt/mTOR | 465.34 | 252917-06-9 | C22H18Cl2N8 |
| Acarbose | in 10mM DMSO | Others | Others | 645.60 | 56180-94-0 | C25H43NO18 |
| Streptozotocin (STZ) | in 10mM DMSO | DNA alkylator | DNA Damage | 265.22 | 18883-66-4 | C8H15N3O7 |
| Glimepiride | in 10mM DMSO | Potassium Channel | Transmembrane Transporters | 490.62 | 93479-97-1 | C24H34N4O5S |
| Resveratrol | in 10mM DMSO | Autophagy | Autophagy | 228.24 | 501-36-0 | C14H12O3 |
| Repaglinide | in 10mM DMSO | Potassium Channel | Transmembrane Transporters | 452.59 | 135062-02-1 | C27H36N2O4 |
| R406 (free base) | in 10mM DMSO | Syk | Angiogenesis | 470.45 | 841290-80-0 | C22H23FN6O5 |
| Saxagliptin | in 10mM DMSO | DPP-4 | Proteases | 315.41 | 361442-04-8 | C18H25N3O2 |
| Dapagliflozin | in 10mM DMSO | SGLT | GPCR & G Protein | 408.87 | 461432-26-8 | C21H25ClO6 |
| Glipizide | in 10mM DMSO | Others | Others | 445.54 | 29094-61-9 | C21H27N5O4S |
| Glyburide (Glibenclamide) | in 10mM DMSO | Potassium Channel | Transmembrane Transporters | 494.00 | 10238-21-8 | C23H28ClN3O5S |
| Gemfibrozil | in 10mM DMSO | PPAR | Metabolism | 250.33 | 25812-30-0 | C15H22O3 |
| Ramipril | in 10mM DMSO | RAAS | Endocrinology & Hormones | 416.51 | 87333-19-5 | C23H32N2O5 |
| Fenofibrate | in 10mM DMSO | PPAR | Metabolism | 360.83 | 49562-28-9 | C20H21ClO4 |
| AICAR (Acadesine) | in 10mM DMSO | AMPK | PI3K/Akt/mTOR | 258.23 | 2627-69-2 | C9H14N4O5 |
| Curcumin | in 10mM DMSO | NF-κB,HDAC,Histone Acetyltransferase,Nrf2 | Epigenetics | 368.38 | 458-37-7 | C21H20O6 |
| Daidzein | in 10mM DMSO | Others | Others | 254.24 | 486-66-8 | C15H10O4 |
| Valsartan | in 10mM DMSO | RAAS | Endocrinology & Hormones | 435.52 | 137862-53-4 | C24H29N5O3 |
| Nicotinamide (Vitamin B3) | in 10mM DMSO | Sirtuin | DNA Damage | 122.12 | 98-92-0 | C6H6N2O |
| Metformin HCl | in 10mM DMSO | Autophagy | Autophagy | 165.62 | 1115-70-4 | C4H12ClN5 |
| Pioglitazone HCl | in 10mM DMSO | P450 (e.g. CYP17) | Metabolism | 392.90 | 112529-15-4 | C19H21ClN2O3S |
| Rosiglitazone HCl | in 10mM DMSO | PPAR | Metabolism | 393.89 | 302543-62-0 | C18H20ClN3O3S |
| Roflumilast | in 10mM DMSO | PDE | Metabolism | 403.21 | 162401-32-3 | C17H14Cl2F2N2O3 |
| GSK1292263 | in 10mM DMSO | GPR | Endocrinology & Hormones | 456.56 | 1032823-75-8 | C23H28N4O4S |
| LY2608204 | in 10mM DMSO | Others | Others | 559.81 | 1234703-40-2 | C28H37N3O3S3 |
| R406 | in 10mM DMSO | FLT3,Syk | Angiogenesis | 628.63 | 841290-81-1 | C28H29FN6O8S |
| Bilobalide | in 10mM DMSO | Others | Others | 326.30 | 33570-04-6 | C15H18O8 |
| Kaempferol | in 10mM DMSO | Fatty Acid Synthase,Estrogen/progestogen Receptor | Endocrinology & Hormones | 286.23 | 520-18-3 | C15H10O6 |
| Luteolin | in 10mM DMSO | PDE | Metabolism | 286.24 | 491-70-3 | C15H10O6 |
| Morin Hydrate | in 10mM DMSO | Others | Others | 320.25 | 6202-27-3 | C15H12O8 |
| Phlorizin | in 10mM DMSO | SGLT | GPCR & G Protein | 436.41 | 60-81-1 | C21H24O10 |
| Rutin | in 10mM DMSO | Immunology & Inflammation related | Immunology & Inflammation | 610.52 | 153-18-4 | C27H30O16 |
| Yohimbine HCl | in 10mM DMSO | Adrenergic Receptor | Neuronal Signaling | 390.90 | 65-19-0 | C21H27ClN2O3 |
| 5-hydroxytryptophan (5-HTP) | in 10mM DMSO | Others | Others | 220.22 | 56-69-9 | C11H12N2O3 |
| Naringenin | in 10mM DMSO | P450 (e.g. CYP17) | Metabolism | 272.25 | 480-41-1 | C15H12O5 |
| Tolbutamide | in 10mM DMSO | Potassium Channel | Transmembrane Transporters | 270.35 | 64-77-7 | C12H18N2O3S |
| Bethanechol chloride | in 10mM DMSO | AChR | Neuronal Signaling | 196.68 | 590-63-6 | C7H17ClN2O2 |
| Nateglinide | in 10mM DMSO | Potassium Channel | Transmembrane Transporters | 317.42 | 105816-04-4 | C19H27NO3 |
| Rosiglitazone maleate | in 10mM DMSO | PPAR | Metabolism | 473.50 | 155141-29-0 | C22H23N3O7S |
| Phenformin HCl | in 10mM DMSO | AMPK | PI3K/Akt/mTOR | 241.72 | 834-28-6 | C10H16ClN5 |
| Rosiglitazone | in 10mM DMSO | PPAR | Metabolism | 357.43 | 122320-73-4 | C18H19N3O3S |
| Miglitol | in 10mM DMSO | Others | Others | 207.22 | 72432-03-2 | C8H17NO5 |
| Pioglitazone | in 10mM DMSO | PPAR | Metabolism | 356.44 | 111025-46-8 | C19H20N2O3S |
| Gliclazide | in 10mM DMSO | Potassium Channel | Transmembrane Transporters | 323.41 | 21187-98-4 | C15H21N3O3S |
| Inulin | in 10mM DMSO | Others | Others | N/A | 9005-80-5 | C12H22O11R |
| Fostamatinib (R788) | in 10mM DMSO | Syk | Angiogenesis | 580.46 | 901119-35-5 | C23H26FN6O9P |
| Fasiglifam?(TAK-875) | in 10mM DMSO | GPR | Endocrinology & Hormones | 533.63 | 1374598-80-7 | C58H66O15S2 |
| WAY-100635 Maleate | in 10mM DMSO | 5-HT Receptor | Neuronal Signaling | 538.64 | 1092679-51-0 | C29H38N4O6 |
| CP-91149 | in 10mM DMSO | Phosphorylase | Metabolism | 399.87 | 186392-40-5 | C21H22ClN3O3 |
| Canagliflozin | in 10mM DMSO | SGLT | GPCR & G Protein | 444.52 | 842133-18-0 | C24H25FO5S |
| Alogliptin（SYK-322）benzoate | in 10mM DMSO | DPP-4 | Proteases | 461.51 | 850649-62-6 | C18H21N5O2 |
| CHIR-99021 (CT99021) HCl | in 10mM DMSO | GSK-3 | PI3K/Akt/mTOR | 501.80 | 1797989-42-4 | C22H19Cl3N8 |
| Linagliptin | in 10mM DMSO | DPP-4 | Proteases | 472.54 | 668270-12-0 | C25H28N8O2 |
| Vildagliptin (LAF-237) | in 10mM DMSO | DPP-4 | Proteases | 303.40 | 274901-16-5 | C17H25N3O2 |
| Sodium salicylate | in 10mM DMSO | NF-κB | NF-κB | 161.11 | 54-21-7 | C7H6NaO3 |
| Gliquidone | in 10mM DMSO | Potassium Channel | Transmembrane Transporters | 527.63 | 33342-05-1 | C27H33N3O6S |
| Tauroursodeoxycholic Acid (TUDCA) | in 10mM DMSO | Others | Others | 499.70 | 14605-22-2 | C26H45NO6S |
| Elafibranor | in 10mM DMSO | PPAR | Metabolism | 384.49 | 923978-27-2 | C22H24O4S |
| Cinnamaldehyde | in 10mM DMSO | TRPV | Transmembrane Transporters | 132.16 | 14371-10-9 | C9H8O |
| Isoferulic Acid | in 10mM DMSO | Others | Others | 194.18 | 537-73-5 | C10H10O4 |
| Neomangiferin | in 10mM DMSO | Others | Others | 584.48 | 64809-67-2 | C25H28O16 |
| Mangiferin | in 10mM DMSO | Others | Others | 422.34 | 4773-96-0 | C19H18O11 |
| Amentoflavone | in 10mM DMSO | Others | Others | 538.46 | 1617-53-4 | C30H18O10 |
| Astaxanthin | in 10mM DMSO | Others | Others | 596.84 | 472-61-7 | C40H52O4 |
| Loganin | in 10mM DMSO | BACE,AChR | Neuronal Signaling | 390.38 | 18524-94-2 | C17H26O10 |
| 1-Deoxynojirimycin | in 10mM DMSO | Others | Others | 163.17 | 19130-96-2 | C6H13NO4 |
| Iso-Steviol | in 10mM DMSO | Others | Others | 318.45 | 27975-19-5 | C20H30O3 |
| Swertiamarin | in 10mM DMSO | Others | Others | 374.34 | 17388-39-5 | C16H22O10 |
| Acetyl Resveratrol | in 10mM DMSO | Sirt12 | others | 354.35 | 42206-94-0 | C20H18O6 |
| (+)-Catechin hydrate | in 10mM DMSO | Others | Others | 290.27 | 225937-10-0 | C15H14O6 |
| Sitagliptin phosphate monohydrate | in 10mM DMSO | DPP-4 | Proteases | 523.32 | 654671-77-9 | C16H20F6N5O6P |
| Voglibose | in 10mM DMSO | Others | Others | 267.28 | 83480-29-9 | C10H21NO7 |
| Chlorpropamide | in 10mM DMSO | Others | Others | 276.74 | 94-20-2 | C10H13ClN2O3S |
| Bromocriptine Mesylate | in 10mM DMSO | Others | Others | 750.70 | 22260-51-1 | C32H40BrN5O5.CH4O3S |
| Tyloxapol | in 10mM DMSO | Others | Others |  | 25301-02-4 | C51H80O6R5 |
| Diflunisal | in 10mM DMSO | COX | Neuronal Signaling | 250.20 | 22494-42-4 | C13H8F2O3 |
| Teneligliptin hydrobromide | in 10mM DMSO | DPP-4 | Proteases | 628.86 | 906093-29-6 | C22H30N6ORS |
| Saxagliptin hydrate | in 10mM DMSO | DPP-4 | Proteases | 333.43 | 945667-22-1 | C18H27N3O3 |
| Saccharin | in 10mM DMSO | Others | Others | 183.18 | 81-07-2 | C7H5NO3S |
| Fingolimod (FTY720) HCl | in 10mM DMSO | S1P Receptor | GPCR & G Protein | 343.90 | 162359-56-0 | C19H34ClNO2 |
| Trelagliptin succinate | in 10mM DMSO | DPP-4 | Proteases | 475.47 | 1029877-94-8 | C22H26FN5O6 |
| Sitagliptin | in 10mM DMSO | DPP-4 | Proteases | 407.31 | 486460-32-6 | C16H15F6N5O |
| Proanthocyanidins | in 10mM DMSO | Others | Others | 594.52 | 20347-71-1 | C30H26O13 |
| Taurocholic acid sodium salt hydrate | in 10mM DMSO | Others | Others | 537.68 | 345909-26-4 |  |
| Thymol | in 10mM DMSO | Immunology & Inflammation related | Immunology & Inflammation | 150.22 | 89-83-8 | C10H14O |
| Fructose | in 10mM DMSO | Others | Others | 180.16 | 57-48-7 | C6H12O6 |
| Alogliptin | in 10mM DMSO | DPP-4 | Proteases | 339.39 | 850649-61-5 | C18H21N5O2 |
| D-Tagatose | in 10mM DMSO | Others | Others | 180.16 | 87-81-0 | C6H12O6 |
| Ertugliflozin | in 10mM DMSO | SGLT2 | Ion-Channel | 436.88 | 1210344-57-2 | C22H25ClO7 |
| Dapagliflozin propanediol monohydrate | in 10mM DMSO | SGLT | GPCR & G Protein | 502.98 | 960404-48-2 | C24H35ClO9 |
| NSI-189 | in 10mM DMSO | Others | Others | 366.50 | 1270138-40-3 | C22H30N4O |
| Acetohexamide | in 10mM DMSO | Others | Others | 324.40 | 968-81-0 | C15H20N2O4S |
| Canagliflozin hemihydrate | in 10mM DMSO | SGLT | GPCR & G Protein | 907.05 | 928672-86-0 | C48H52F2O11S2 |
| Anagliptin | in 10mM DMSO | DPP-4 | Proteases | 383.45 | 739366-20-2 | C19H25N7O2 |
| Zebularine | in 10mM DMSO | DNA Methyltransferase | Epigenetics | 228.20 | 3690-10-6 | C9H12N2O5 |
| JTE 013 | in 10mM DMSO | S1P Receptor | GPCR & G Protein | 408.29 | 383150-41-2 | C17H19Cl2N7O |
| PF-04620110 | in 10mM DMSO | Transferase | Metabolism | 396.44 | 1109276-89-2 | C21H24N4O4 |
| Alvelestat (AZD9668) | in 10mM DMSO | Serine Protease | Proteases | 545.53 | 848141-11-7 | C24H20F3N5O4S |
| Apabetalone (RVX-208) | in 10mM DMSO | Epigenetic Reader Domain | Epigenetics | 370.40 | 1044870-39-4 | C20H22N2O5 |
| AdipoRon | in 10mM DMSO | AdipoR | Metabolism | 428.52 | 924416-43-3 | C27H28N2O3 |
| WS6 | in 10mM DMSO | IκB/IKK | NF-κB | 568.59 | 1421227-53-3 | C29H31F3N6O3 |
| Trelagliptin | in 10mM DMSO | DPP-4 | Proteases | 357.38 | 865759-25-7 | C18H20FN5O2 |
| VGX-1027 | in 10mM DMSO | Immunology & Inflammation related | Immunology & Inflammation | 205.21 | 6501-72-0 | C11H11NO3 |
| AZD7545 | in 10mM DMSO | Others | Others | 478.87 | 252017-04-2 | C19H18ClF3N2O5S |
| Piperlongumine | in 10mM DMSO | ROS,CRM1 | Immunology & Inflammation | 317.34 | 20069-09-4 | C17H19NO5 |
| Obeticholic Acid | in 10mM DMSO | FXR | Others | 420.63 | 459789-99-2 | C26H44O4 |
| SRT2104 (GSK2245840) | in 10mM DMSO | Sirtuin | DNA Damage | 516.64 | 1093403-33-8 | C26H24N6O2S2 |
| GW9508 | in 10mM DMSO | GPR | Endocrinology & Hormones | 347.41 | 885101-89-3 | C22H21NO3 |
| GW0742 | in 10mM DMSO | PPAR | Metabolism | 471.49 | 317318-84-6 | C21H17F4NO3S2 |
| Empagliflozin (BI 10773) | in 10mM DMSO | SGLT | GPCR & G Protein | 450.91 | 864070-44-0 | C23H27ClO7 |
| GSK3787 | in 10mM DMSO | PPAR | Metabolism | 392.78 | 188591-46-0 | C15H12ClF3N2O3S |
| Bardoxolone Methyl | in 10mM DMSO | IκB/IKK | NF-κB | 505.69 | 218600-53-4 | C32H43NO4 |
| Sotagliflozin (LX4211) | in 10mM DMSO | SGLT | GPCR & G Protein | 424.94 | 1018899-04-1 | C21H25ClO5S |
| BI-78D3 | in 10mM DMSO | JNK | MAPK | 379.37 | 883065-90-5 | C13H9N5O5S2 |
| Etomoxir (Na salt) | in 10mM DMSO | Transferase | Metabolism | 320.74 | 828934-41-4 | C15H18ClNaO4 |
| SRT2183 | in 10mM DMSO | Sirtuin | DNA Damage | 468.57 | 1001908-89-9 | C27H24N4O2S |
| MLR-1023 | in 10mM DMSO | Src | Protein Tyrosine Kinase | 202.21 | 41964-07-2 | C11H10N2O2 |
| BGP-15 2HCl | in 10mM DMSO | PARP | DNA Damage | 351.27 | 66611-37-8 | C14H24Cl2N4O2 |
| Troglitazone (CS-045) | in 10mM DMSO | PPAR | Metabolism | 441.54 | 97322-87-7 | C24H27NO5S |
| SRT3025 HCl | in 10mM DMSO | Sirtuin | DNA Damage | 606.20 | 2070015-26-6 | C31H32ClN5O2S2 |
| SR-18292 | in 10mM DMSO | Others | Others | 366.50 | 2095432-55-4 | C23H30N2O2 |
| Tofogliflozin(CSG 452) | in 10mM DMSO | SGLT | GPCR & G Protein | 404.45 | 1201913-82-7 | C22H28O7 |
| Omarigliptin (MK-3102) | in 10mM DMSO | DPP-4 | Proteases | 398.43 | 1226781-44-7 | C17H20F2N4O3S |
| Ipragliflozin (ASP1941) | in 10mM DMSO | SGLT | GPCR & G Protein | 404.45 | 761423-87-4 | C21H21FO5S |
| ACY-738 | in 10mM DMSO | HDAC | Epigenetics | 270.29 | 1375465-91-0 | C14H14N4O2 |
| kira6 | in 10mM DMSO | Others | Others | 518.53 | 1589527-65-0 | C28H25F3N6O |
| Verinurad (RDEA3170) | in 10mM DMSO | Others | Others | 348.42 | 1352792-74-5 | C20H16N2O2S |
| Lanifibranor(IVA-337) | in 10mM DMSO | PPAR | Metabolism | 434.92 | 927961-18-0 | C19H15ClN2O4S2 |
| Maslinic acid | in 10mM DMSO | DNA polymerase B | Cancer | 472.70 | 4373-41-5 | C30H48O4 |
| Sweroside | in 10mM DMSO | Others | Others | 358.34 | 14215-86-2 | C16H22O9 |
| Chicoric acid | in 10mM DMSO | Infection | Others | 474.37 | 70831-56-0 | C22H18O12 |
| Engeletin | in 10mM DMSO | Infection | NF-κB | 434.39 | 572-31-6 | C21H22O10 |
| Alisol A | in 10mM DMSO | Others | Others | 490.72 | 19885-10-0 | C30H50O5 |
| Sequoyitol | in 10mM DMSO | Others | Others | 194.18 | 523-92-2 | C7H14O6 |
| Semaglutide | in 10mM DMSO | Others | Others | 4113.58 | 99291-20-0 | C187H291N45O59 |
| Madecassic acid | in 10mM DMSO | Immunology & Inflamm | Immunology & Inflammation | 504.70 | 18449-41-7 | C6H12O5 |
| Morin | in 10mM DMSO | Others | Cancer | 302.24 | 480-16-0 | C15H10O7 |
| isoleucine | in 10mM Water | Others | Others | 131.17 | 73-32-5 | C6H13NO2 |
| L-Leucine | in 10mM Water | Others | Others | 131.17 | 61-90-5 | C6H13NO2 |
| Hydroxychloroquine Sulfate | in 10mM Water | Autophagy | Autophagy | 433.95 | 747-36-4 | C18H28ClN3O5S |
| Eprodisate disodium | in 10mM Water | Others | Others | 248.19 | 36589-58-9 | C3H6Na2O6S2 |
| 4-Aminobutyric acid | in 10mM Water | GABA Receptor | Neuronal Signaling | 103.12 | 56-12-2 | C4H9NO2 |
| Methyldopa | in 2mM DMSO | 5-HT Receptor,Adrenergic Receptor,Dopamine Receptor,Decarboxylase | Metabolism | 211.21 | 555-30-6 | C10H13NO4 |
| R788 (Fostamatinib) Disodium | in 2mM DMSO | Syk | Angiogenesis | 624.42 | 1025687-58-4 | C23H24FN6Na2O9P |
| Cetilistat | in 2mM DMSO | Lipase | Metabolism | 401.58 | 282526-98-1 | C25H39NO3 |
| Coptisine chloride | in 2mM DMSO | Others | Others | 355.77 | 6020-18-4 | C19H14ClNO4 |

**SUPPLEMENTAL METHODS**

**Single-cell sequencing samples collection**

Aortic valve samples were isolated immediately post-operation and washed with cold 1×PBS, followed by mechanical dissociation using fine scissors. Tissue fragments were digested in DMEM containing collagenase type I (2 mg/mL; Sigma-Aldrich, Saint Louis, MO) to generate single-cell suspensions. The resulting suspensions were transported on ice to BGI Wuhan Research Center for single-cell sequencing. Cell viability was maintained at ≥90%.

**Baseline presentation**

Baseline characteristics were stratified by CKD status. Categorical variables were expressed as number (percentage), and continuous variables as median (quartile).

Associations between creatinine, uric acid, urea nitrogen, and aortic valve SIRT1 expression were evaluated using fully adjusted linear models.

The relationship between CKD stage, creatinine levels, and aortic stenosis risk in the UK Biobank cohort was analyzed using ggpubr (v0.6.0) and plotRCS (v0.1.5).

**Genome-wide association study**

UK Biobank participants were genotyped using the UK BiLEVE and UK Biobank Axiom arrays, yielding 805,426 markers (GRCh37). GWAS analyses were conducted using PLINK 2.0.

Analyses were restricted to unrelated White British individuals, adjusting for age, sex, and the first 10 principal components. Approximately 3% of participants were excluded due to insufficient genotyping quality.

**Mendelian randomization analysis**

Mendelian randomization (MR) analyses were performed using the TwoSampleMR R package (v0.6.4). Instrumental variables (IVs) were selected with: P ≤ 1×10⁻³ in exposure and P ≥ 1×10⁻³ in outcome Palindromic variants were removed. Weak instruments (F < 10) were excluded. Primary analysis used inverse-variance weighted (IVW) models. Additional methods included MR-Egger, weighted median, weighted mode, and maximum likelihood approaches. Sensitivity analyses assessed pleiotropy (MR-Egger intercept) and heterogeneity (Cochran’s Q). To evaluate the causal role of *SIRT1* expression, eQTLs from GTEx v8 were used. Variants with MAF ≥ 0.01 were selected and pruned for LD (r² < 0.9), followed by LD-aware MR analysis.

**Establishment of the CKD mouse model**

A two-step 5/6 nephrectomy model was used. Briefly, mice underwent partial resection of the left kidney followed by total removal of the right kidney one week later. Sham-operated mice underwent identical procedures without resection.

After recovery, mice were fed a high-phosphate diet for 12 weeks. Echocardiographic measurements (Vevo 1100 system) were performed before sacrifice. Blood and tissue samples were collected and stored for downstream analyses.

**Compound treatment strategy**

MCC950 was administered at a dose of 10 mg/kg via weekly tail vein injection. Semaglutide (purchased from Master of Bioactive Molecules) was delivered in a similar manner at a dose of 60 μg/kg/day by subcutaneous injection for one month. For resveratrol intervention, resveratrol was incorporated into the chow at a concentration of 0.04% and provided continuously throughout the experimental period. All injections were performed under standard sterile conditions.

**Construction of a mouse model for tissue-specific SIRT1 intervention**

*Sirt1^flox/flox^* (Strain #008041), *Sirt1*^+/−^ (#026009), and *Sirt1^Tg^* (#013080) mice were obtained from Jackson Laboratory. Tagln-Cre mice were used to generate smooth muscle/myofibroblast-specific SIRT1-modified models. To establish experimental cohorts, *Sirt1^+/−^* or *Sirt1^Tg^* mice were crossed with *Apoe^-/-^* mice to generate *Apoe^-/-^ Sirt1*^+/−^ and *Apoe^-/-^ Sirt1^Tg^* mice, respectively. In parallel, *Sirt1^flox/flox^* mice were crossed with *Apoe^-/-^* mice and further bred with Tagln-Cre mice to generate *Apoe^-/-^* *Sirt1^flox/flox^* Tagln-Cre mice, enabling tissue-specific manipulation of SIRT1 expression.

**Chemicals and reagents**

SRT1720 (catalog#S1129), MCC950 (catalog#S8930), BAY11-7082 (catalog#S2913), and resveratrol (catalog#S1396) were purchased from Selleck (Houston, TX). Semaglutide was purchased from Master of Bioactive Molecules. Lipofectamine 3000 and other transfection-related reagents were purchased from Thermo Fisher Scientific (Waltham, MA). The collagenase type I (SCR103) and Von Kossa staining kit were purchased from Sigma-Aldrich Chemical Co (St. Louis, MO). Specific small interfering RNA (siRNA) for human SIRT1 and NLRP3, scrambled siRNA were purchased from RiBo biotechnology (Guangzhou, China). Alizarin red staining kit (catalog #0223) was purchased from ScienCell Research Laboratories (Carlsbad, CA). ALP activity staining kit (catalog #P0321S) and β-galactosidase Staining Kit (catalog #RG0039) were purchased from Beyotime (Shanghai, China). RIPA Lysis and Extraction Buffer were purchased from Biorad (Hercules, CA). The following antibodies were used for western blot: anti-SIRT1 (Abcam, ab110304, 1:1000 dilution), anti-NLRP3 (Abcam, ab214185, 1:1000 dilution), anti-RUNX2 (Abcam, ab23981, 1:1000 dilution), anti-ALP (Abcam, ab83259, 1:1000 dilution), anti-GAPDH (Proteintech, 60004-1-Ig 1:1000 dilution), anti-IκBα (CST, 4814s, 1:1000 dilution), anti-phospho-IκBα (CST, 2859s, 1:1000 dilution), anti-NF-κB p65 (CST, 8242, 1:1000 dilution), and anti-phospho-NF-κB p65 (CST, 3033, 1:1000 dilution). The following antibodies were used for immunoflourescence: anti-SIRT1 (Abcam, ab110304, 1:100 dilution), anti-NLRP3 (Abcam, ab4207, 1:200 dilution), anti-RUNX2 (Abcam, ab23981, 1:200 dilution), anti-p16 (Santa Cruz Biotechnology, sc-1661, 1:200 dilution), and anti-phospho-NF-κB p65 (CST, 3033, 1:200 dilution). Hyperphosphate diet was purchased from SYSE Bio-tech. Co., Ltd (Changzhou, China). Other cell culture reagents including Dulbecco′s modified Eagle′s medium (DMEM), streptomycin, fetal bovine serum (FBS), and penicillin were purchased from Gibco (Amarillo, TX).

**RNA interference**

VICs were transfected with siRNA targeting SIRT1 or NLRP3 using Lipofectamine 3000. Knockdown efficiency was confirmed by western blot 72 h post-transfection.

**Protein extraction and Western blot**

Proteins were extracted using RIPA buffer with protease/phosphatase inhibitors. Protein concentrations were measured by BCA assay.

Samples were separated by SDS-PAGE, transferred to PVDF membranes, and incubated with primary antibodies overnight at 4°C. Detection was performed using ECL and analyzed with ImageJ.

**ELISA**

Serum levels of IL-1β, IL-18, TNF-α, MCP-1, and IL-6 were measured using commercial ELISA kits according to manufacturers’instructions.

**Immunofluorescence staining**

Human paraffin sections and mouse cryosections (5 μm) were prepared and stained with primary antibodies overnight at 4°C, followed by fluorescent secondary antibodies.

For cultured VICs, cells were fixed, permeabilized, and stained similarly. Nuclei were counterstained with DAPI. Imaging was performed using confocal microscopy (FV3000, Olympus).

**Von kossa staining**

Sections were incubated with 5% silver nitrate, exposed to UV light, and counterstained. Calcified areas were quantified using Image-Pro Plus software.

**Alizarin Red staining**

After 21 days of osteogenic induction, cells were fixed and stained with 2% Alizarin Red. Mineralization was quantified using Image-Pro Plus.

**Alkaline Phosphatase staining**

Cells were stained using BCIP/NBT substrate after 10 days of induction. Positive staining was quantified.

**SA-β-gal Staining**

Senescence-associated β-galactosidase staining was performed, and positive cells were quantified microscopically.

**Anti-diabetic compound library screening**

A 96-well anti-diabetic compound library (Selleck, L2900) was screened in VICs under high-phosphate conditions.

Cells were treated with compounds (10 nM), and SIRT1/NLRP3 expression was assessed using high-content confocal imaging. Fifteen non-overlapping fields per well were analyzed, with three replicates per compound. The full compound list is provided in **Supplemental eTable 4**.
